# Sequence and Role in Virulence of the Three Plasmid Complement of the Model Tumor-Inducing Bacterium *Pseudomonas savastanoi* pv. savastanoi NCPPB 3335

**DOI:** 10.1371/journal.pone.0025705

**Published:** 2011-10-11

**Authors:** Leire Bardaji, Isabel Pérez-Martínez, Luis Rodríguez-Moreno, Pablo Rodríguez-Palenzuela, George W. Sundin, Cayo Ramos, Jesús Murillo

**Affiliations:** 1 Departamento de Producción Agraria, Escuela Técnica Superior de Ingenieros Agrónomos, Universidad Pública de Navarra, Pamplona, Spain; 2 Área de Genética, Facultad de Ciencias, Universidad de Málaga, Instituto de Hortofruticultura Subtropical y Mediterránea “La Mayora,” Málaga, Spain; 3 Centro de Biotecnología y Genómica de Plantas, Escuela Técnica Superior de Ingenieros Agrónomos, Universidad Politécnica de Madrid, Campus de Montegancedo, Pozuelo de Alarcón, Madrid, Spain; 4 Department of Plant Pathology and Center for Microbial Ecology, Michigan State University, East Lansing, Michigan, United States of America; University of Wisconsin-Milwaukee, United States of America

## Abstract

*Pseudomonas savastanoi* pv. savastanoi NCPPB 3335 is a model for the study of the molecular basis of disease production and tumor formation in woody hosts, and its draft genome sequence has been recently obtained. Here we closed the sequence of the plasmid complement of this strain, composed of three circular molecules of 78,357 nt (pPsv48A), 45,220 nt (pPsv48B), and 42,103 nt (pPsv48C), all belonging to the pPT23A-like family of plasmids widely distributed in the *P. syringae* complex. A total of 152 coding sequences were predicted in the plasmid complement, of which 38 are hypothetical proteins and seven correspond to putative virulence genes. Plasmid pPsv48A contains an incomplete Type IVB secretion system, the type III secretion system (T3SS) effector gene *hopAF1*, gene *ptz*, involved in cytokinin biosynthesis, and three copies of a gene highly conserved in plant-associated proteobacteria, which is preceded by a *hrp* box motif. A complete Type IVA secretion system, a well conserved origin of transfer (*oriT*), and a homolog of the T3SS effector gene *hopAO1* are present in pPsv48B, while pPsv48C contains a gene with significant homology to isopentenyl-diphosphate delta-isomerase, type 1. Several potential mobile elements were found on the three plasmids, including three types of MITE, a derivative of IS*801*, and a new transposon effector, IS*Psy30*. Although the replication regions of these three plasmids are phylogenetically closely related, their structure is diverse, suggesting that the plasmid architecture results from an active exchange of sequences. Artificial inoculations of olive plants with mutants cured of plasmids pPsv48A and pPsv48B showed that pPsv48A is necessary for full virulence and for the development of mature xylem vessels within the knots; we were unable to obtain mutants cured of pPsv48C, which contains five putative toxin-antitoxin genes.

## Introduction

The gamma proteobacterium *Pseudomonas savastanoi* pv. savastanoi causes olive (*Olea europaea* L.) knot disease, one of the most economically relevant diseases of the olive crop [1]. *P. savastanoi* pv. savastanoi is part of the *P. syringae* complex, which includes at least 10 *Pseudomonas* species and 60 pathovars of *P. syringae*, most of which are pathogenic to plants, and whose taxonomy is confusing and still unresolved [2], [3], [4]. Indeed, DNA-DNA hybridization studies indicate that the *P. syringae* complex could be split in nine different genomospecies [2]. In this scheme, *P. savastanoi* pv. savastanoi has been assigned to the species *P. amygdali* (genomospecies 2) together with 16 other *P. syringae* pathovars, including *P. syringae* pv. aesculi, glycinea, phaseolicola and tabaci, whose genomes were recently sequenced [5], [6], [7], [8], [9]. The majority of pathovars from the *P. syringae* complex cause foliar necrosis in a large diversity of herbaceous hosts, including the model plant *Arabidopsis*, and are divided into pathovars depending of their particular host range [3]. Only a few pathovars infect woody hosts, such as pvs. aesculi and morsprunorum, infecting the vascular system and producing trunk lesions or causing foliar or flower necroses. *P. savastanoi* pv. savastanoi also infects woody hosts, but it is significant in that it is one of a few closely-related pathovars that cause aerial tumors in their plant hosts. Infection of olive by *P. savastanoi* pv. savastanoi results in overgrowth formation on the stems and branches, and rarely on the leaves and fruits. The disease is considered to reduce both olive yield and productivity [10], [11], and few commercial cultivars are significantly tolerant to olive knot disease [12].

*P. savastanoi* pv. savastanoi strain NCPPB 3335, isolated in France from a diseased olive tree, is being used as a model organism, mostly because of its ability to accept foreign DNA with a high frequency [13] and its capability of inducing olive knots in young micropropagated olive plants [14], [15], a model system that has recently allowed a description of the endopathogenic lifestyle of this bacterium in olive knots [15]. Analysis of the NCPPB 3335 draft genome has identified various features that could contribute to the ability of this strain to survive in a woody host, including genes related with the transport and catabolism of plant-derived aromatic compounds, the duplication of sequences related with well-known pathogenicity and virulence factors such as those involved in the biosynthesis of the phytohormone indoleacetic acid, and the inventory of strain-specific putative type III secretion system (T3SS) effectors [16].

Most strains of the *P. syringae* complex, regardless of pathovar, contain at least one indigenous plasmid that belongs to the pPT23A plasmid family, a group of plasmids that share the major replication gene *repA* [17], [18], [19], [20], [21]. pPT23A-family plasmids (PFPs) typically encode determinants that contribute to ecological fitness *in planta* of their phytopathogenic bacterial host. These determinants can include T3SS effectors or phytotoxin biosynthetic genes that contribute to virulence and other determinants such as the UV radiation tolerance genes *rulAB* that contribute to increased survival on sunlight-exposed plant surfaces [22], [23], [24], [25]. In addition, many PFPs are capable of horizontal transfer, and retrospective comparative sequence analyses have suggested that most PFPs are mosaics and comprise gene collections that have been obtained via horizontal transfer by their respective bacterial host [20].

Strains of *P. savastanoi* pv. savastanoi typically harbor between one and four PFPs, and sometimes also contain non-PFP plasmids [21]. The gene complement of *P. savastanoi* pv. savastanoi plasmids includes phytohormone biosynthetic genes, T3SS effectors, two distinct type IV secretion systems, and multiple insertion elements [21]. Several indigenous plasmids from *P. savastanoi* pv. savastanoi have been shown to contribute to virulence and to competitive fitness of this pathogen [21], [26], [27], [28], [29].

Determination of complete, closed plasmid sequences from phytopathogens has contributed significantly to our understanding of the origin and evolution of these molecules, and of their role in plant pathogenesis [24]. The *P. savastanoi* pv. savastanoi – olive model represents an excellent woody host pathosystem from which to study the role of plasmid-encoded genes in pathogenesis. We hypothesized that determination of the complete sequence of the plasmid complement of *P. savastanoi* pv. savastanoi NCPPB 3335 would facilitate genetic studies detailing the role of these plasmids in pathogenesis and tumor formation on olive. In this study, we report the sequence and detailed analysis of three plasmids (42, 45, and 78 kb) from this strain, as well as the evaluation of the role of individual plasmids in virulence.

## Results

### Identification and sequencing of the native plasmid complement of *P. savastanoi* pv. savastanoi NCPPB 3335

**Figure 1:**
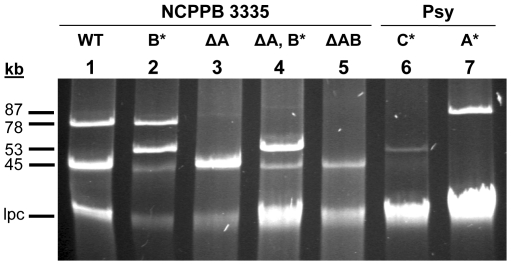
Derivatives of *P. savastanoi* pv. savastanoi strain NCPPB 3335 (syn. Psv48) and *P. syringae* pv. syringae strain B728a obtained by plasmid tagging and curing. Uncut plasmids were separated by electrophoresis in agarose gels. Strain NCPPB 3335 (lane 1, WT) was mutagenized with Tn*5*-GDYN1 (8.8 kb) and insertions in plasmids pPsv48A (lane 7, A*), pPsv48B (lanes 2 and 4, B*) and pPsv48C (lane 6, C*) are evident by a retardation in mobility. Tagging or curing plasmid pPsv48B reveals the presence of plasmid pPsv48C (lanes 2, 4 and 5), which is of similar size but has a lower copy number. From the tagged derivatives, we obtained strains cured of plasmids pPsv48A (lanes 3 and 4, ΔA) and both pPsv48A and pPsv48B (lane 5, ΔAB). Lanes 6 and 7 correspond to strain B728a transformed with mutagenized plasmids pPsv48C and pPsv48A respectively. The molecular weights of the plasmids are indicated in kb to the left; lpc: Linearized plasmid and chromosomal DNA.

Native plasmids from strains of the *P. syringae* group generally share a large amount of repeated sequences [17], [21], [24], and our initial analyses showed that this was the case with the plasmids from strain NCPPB 3335. Therefore, we approached their sequencing by first individualizing them and obtaining derivatives of NCPPB 3335 cured of one or more of the native plasmids; this strategy would also allow us to additionally assess their role in the bacterial life cycle and virulence. We followed a simple strategy that involved tagging individual plasmids with a transposon conferring antibiotic resistance and conditional lethality [30], [31] using the transposon Tn*5*-GDYN1 [32], which contains the levansucrase gene *sacB* and allows for the selection of derivatives cured of the tagged plasmids in media with sucrose. Mutagenesis with Tn*5*-GDYN1 yielded approximately 23% insertions in native plasmids, as deduced from their altered mobility in plasmid profile gels (Figure 1).

We previously identified two native plasmids, pPsv48A (73 kb) and pPsv48B (42 kb), in strain NCPPB 3335 [21]; after mutagenesis, we were able to visualize a new plasmid comigrating with pPsv48B, designated pPsv48C, which had a lower copy number and that was only evident in mutants with a transposon insertion in either plasmid B or plasmid C (Figure 1 and not shown). Plasmids pPsv48A and pPsv48C were successfully transferred to the plasmidless strain *P. syringae* pv. syringae (Psy) B728a; however, pPsv48B could not be transferred intact to this or any other tested pseudomonad strain, such as *P. fluorescens* SBW25 or *P. putida* KT2440, as the plasmid suffered large deletions in the process (not shown). Additionally, it was possible to obtain strains Psv48ΔA and Psv48ΔAB (Figure 1), cured respectively of plasmids pPsv48A and of pPsv48A and pPsv48B. Despite numerous attempts, it was not possible to obtain a derivative of strain NCPPB 3335 cured of plasmid pPsv48C. Likewise, repeated attempts to obtain a derivative cured of pPsv48B by itself resulted in clones containing reorganized plasmid profiles, and it was possible to successfully cure this plasmid only in strains lacking pPsv48A.

**Figure 2:**
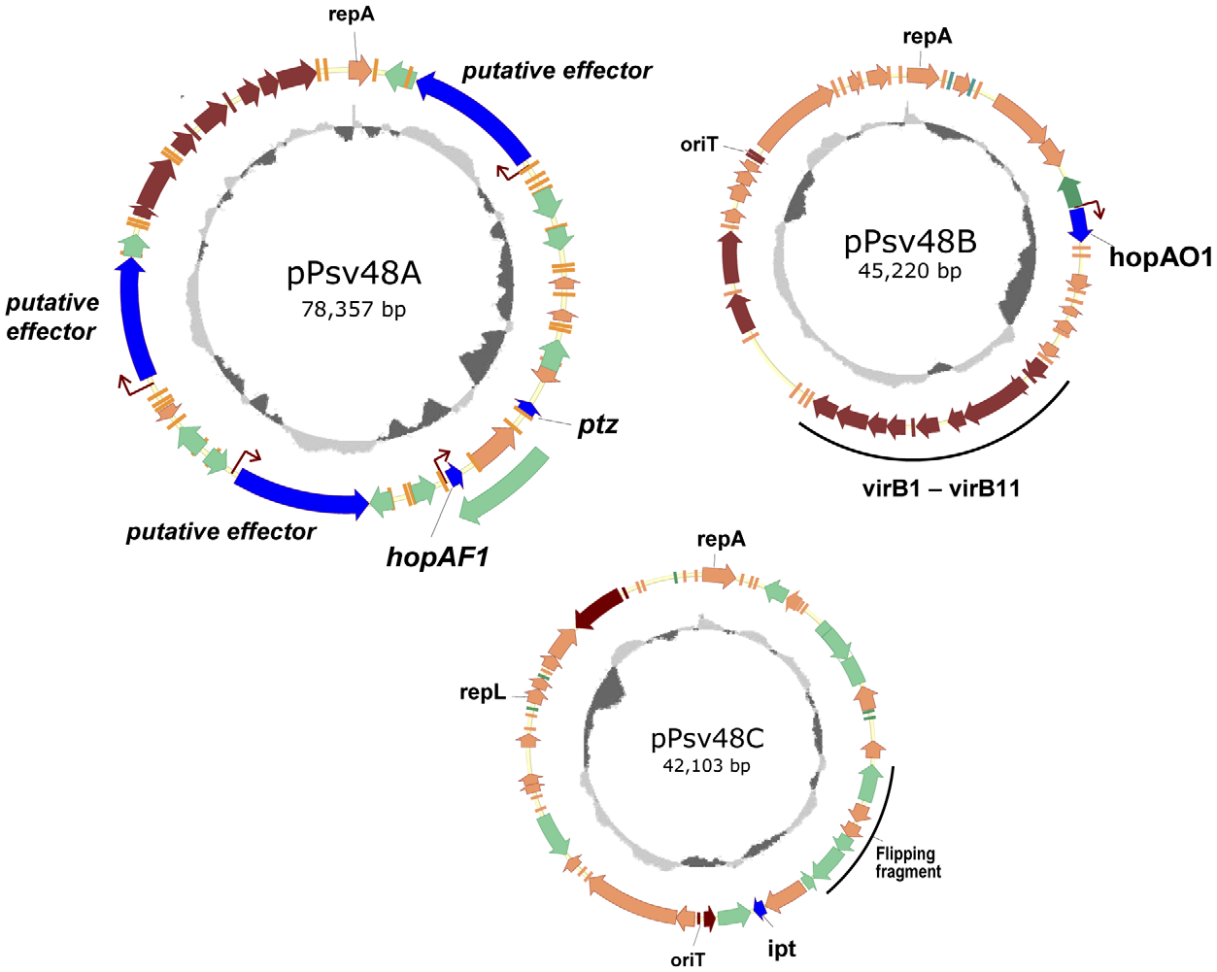
Genetic maps of plasmids pPsv48A, pPsv48B and pPsv48C. Genes are color-coded according to category or putative function as follows: genes putatively involved in host-interaction and virulence are shown in blue; putative mobile elements are indicated in green, and homologs of Type IV secretion systems genes are shown in brown; the remaining genes are shown in orange. The effector transposon IS*Psy30* is shown as a green arrow out of the map of pPsv48A. Prototypical *hrp* boxes are indicated by brown arrows. The inner circles indicate the GC content (window, 1000; step, 5), with values above and below the average shown in light and dark grey, respectively. Plasmids are drawn to scale.

The complete sequence of the plasmids yielded three circular molecules belonging to the pPT23A-like family group with the characteristics summarized in Figure 2 and Table 1. A total of 152 CDSs were predicted for the three plasmids, with the deduced products of half of them assigned to the categories of “hypothetical protein” (38 CDSs) and “DNA metabolism” (37 CDSs) (Tables 1 and S1). Coding capacity is variable and is not related to plasmid size (Table 1); indeed, pPsv48B has the highest density of coding DNA, with 48 CDSs (excluding transposases), whereas pPsv48C only contains 33 CDSs, despite being nearly the same size as pPsv48B. The overall G+C content of the plasmids is close to the 57.12% G+C of the NCPPB 3335 genome, although they contained 22 CDSs with less than 50% (34.4–49.4% G+C) and 8 CDSs higher than 62% (62–65.1% G+C) (Table S2), that could have been acquired via horizontal gene transfer. Among these CDSs there are three putative virulence genes: the type III effector *hopAF1* (47.2% G+C), and two genes putatively involved in the biosynthesis of phytohormones, gene *ipt* (47.7% G+C), a putative isopentenyl-diphosphate delta-isomerase gene, and *ptz* (43.4% G+C), an isopentenyl transferase gene. Analysis of the three plasmids with IslandViewer predicted one genomic island in pPsv48A (6,140 nt; coordinates 21,767–27,906) and another in pPsv48B (4,723 nt; coordinates 11,433–16,155). The first putative island contains CDSs PSPSV_A0019 to PSPSV_A0025, coding for a putative toxin-antitoxin system, three hypothetical proteins and *ptz*. The second island contains CDSs PSPSV_B0011 to PSPSV_B0020, which also code for a putative toxin-antitoxin system and a putative stability/partition system; among others, the putative island contains CDSs coding for a putative bacteriocin immunity protein and a putative transcription antiterminator (see Table S2).

**Table 1:** General characteristics of the three native plasmids of *P. savastanoi* pv. *savastanoi* NCPPB 3335.

			n° ORF		Coding percentage		
Plasmid	Size (nt)	G+C%	Total	Without Tnases^a^	Total	Without Tnases^a^	% ISs^b^
pPsv48A	78,357	57.87	60	49	77.0	64.8	24.8
pPsv48B	45,220	55.66	50	48	81.4	78.6	3.7
pPsv48C	42,103	54.18	42	33	64.7	51.0	29.5

a Tnases, transposases.

b Percentage of the total nucleotide sequence occupied by putative mobile elements.

Plasmid pPsv48B contains 15 CDSs that might constitute a complete Type IVA secretion system (Table S3) and a well conserved origin of transfer (*oriT*), which is also present in pPsv48C. Therefore, it is highly likely that pPsv48B is a conjugative plasmid, whereas pPsv48C might be mobilizable by plasmid pPsv48B. We found an incomplete Type VIB conjugation system in pPsv48A (Table S3), but not an origin of transfer, suggesting that this plasmid might not be mobilizable by conjugation.

We found seven potential virulence genes in the plasmids of NCPPB 3335, five of which are preceded by a HrpL regulatory motif, or *hrp* box (Tables S1 and S4). Plasmid pPsv48A contains a gene involved in cytokinins biosynthesis, *ptz*, and four CDSs preceded by a *hrp* box. These four CDSs included three alleles (PSPSV_A0005, PSPSV_A0035 and PSPSV_A0046) of a highly conserved gene found in many plant-associated proteobacteria and a chimeric allele of effector gene *hopAF1* captured by transposon IS*Psy30* (see below). pPsv48B contains a putative T3SS effector identified as *hopAO1* (sin. *avrPphD2*) which functions as a suppressor of plant resistance triggered by PAMPS [33], and that is preceded by a consensus *hrp* box [34]. pPsv48C contains a CDS with significant homology to isopentenyl-diphosphate delta-isomerase, type 1 (InterPro family IPR011876), which could participate in cytokinins biosynthesis.

The three plasmids contained 11 types of insertion sequences and three miniature inverted-repeats transposable elements (MITE) (Table S5). Among these, plasmid pPsv48A contains a putative effector transposon, designated IS*Psy30*, which has captured a chimeric DNA fragment containing a fragment of the effector gene *hopAY1* (273 nt before the start codon, including a *hrp* box, and the first 18 aa) fused to an allele of *hopAF1* (Figure S1). The 273 nt fragment also includes the 38 nt right inverted repeat of IS*Psy30* in the alleles of *hopAY1* found in strains of *P. syringae* pvs. eriobotryae and phaseolicola (accession no. AB018553, CP000059, and AY603426), suggesting that this fragment might have arrived to *P. syringae* with the effector transposon and then incorporated to effector gene *hopAY1*. Chimeric effectors are very common in animal and plant pathogens, originating from a shuffling process called “terminal reassortment” that favors the rapid emergence of new host specificities [35].

We identified three MITEs, ranging from 0.1 to 0.3 kb and with varying copy numbers (Table S5). MITE*Psy1* (100 nt) is present in many strains of the *P. syringae* group and was originally found altering host range specificity by insertion into the effector gene *avrPphE* [36], and later shown to actively transpose [37]. MITE*Psy2* is 228 nt and probably originated from IS*Psy30* because they have nearly identical terminal inverted repeats [37]. The four full-length copies of MITE*Psy2* present in the three plasmids (Table S5) are nearly identical, and between 90–92% identical to a copy in plasmid p1448A-A from *P. syringae* pv. phaseolicola 1448A. The copy of MITE*Psy2* in pPsv48A is flanked by a direct 5 nt repetition, as it occurs with the IS*Psy30* homolog in plasmid pGNB1 [38], suggesting that it originated from a true transposition event. Finally, the terminal ends of MITE*Psy3* are nearly identical to those of transposon IS*Thsp9*, from *Thiomonas* sp. (Figure S2), and the element is also present in two truncated copies in p1448A-A.

The three plasmids contain a replication initiator protein gene (*repA*) that defines the pPT23A-like family of plasmids typical of the *P. syringae* group [17], [24]. In a phylogenetic analysis of *repA* (Figure S3), the plasmids of strain NCPPB 3335 clustered with diverse plasmids from *P. savastanoi* pv. savastanoi, suggesting that they share a recent common origin, although they were separated from plasmids isolated from other pathovars of the genomospecies 2, including other plasmids from pv. savastanoi, as previously described [20], [39]. The *repA* sequences of the pPsv48B and pPsv48C plasmids cluster tightly together on one branch, which is not surprising given the high identity they share (97.5% amino acid identity), and also closely to the *repA* of pPsv48A.

Plasmid pPsv48C contains an additional putative replication protein, *repL* (PSPSV_C0043), with homologs in *Thiomonas intermedia* K12, *Burkholderia* and enterobacteriaceae. We were unable to demonstrate autonomous replication mediated by *repL*, with or without the accompanying downstream CDS, coding for a putative entry exclusion protein, in either *E. coli* or strains of the *P. syringae* complex. This suggests that *repL* might not contribute to the maintenance of pPsv48C in *P. savastanoi* pv. savastanoi or to its dissemination to the enterobacterial populations that are frequently found in knots or the olive phyllosphere [40].

To evaluate the conservation of the plasmid backbone, we made a comparison of the entire plasmid sequences against the NCBI database. As expected, due to their dynamic nature, the structure of the three plasmids is not fully conserved in any other sequenced plasmid, although they shared variable regions of synteny. pPsv48B most closely resembles the plasmids pPMA4326A (accession no. AY603979), pPSR1 (AY342395) and p1448A-B (CP000060), sharing synteny over approximately 24 to 30 kb that corresponds to the replication region, the Type IVA secretion system genes, and a fragment including genes *mobCB* and a *gntR*-like transcriptional regulator (Figure S4 and Table S3). Conversely, the structure of plasmids A and C is poorly conserved, with only stretches smaller than 10 kb found in other plasmids. Nevertheless, a total of around 10 kb of pPsv48C shares at least 80% nucleotide identity with pPsv48B, including 2.5 kb surrounding gene *repA* (Figure S4).

### Virulence on olive plants of plasmid-cured derivatives of strain NCPPB 3335

**Figure 3:**
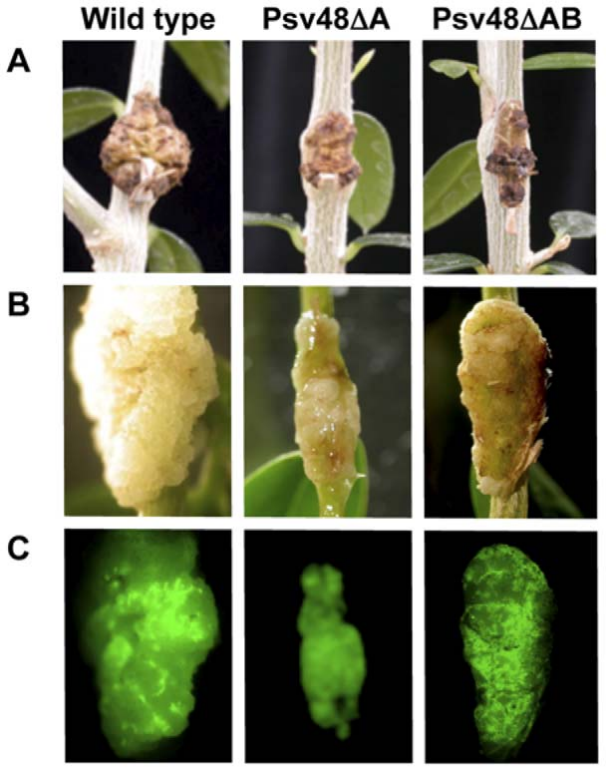
Symptoms induced by derivatives of strain NCPPB 3335 cured of native plasmids. (**A**) Symptoms induced on the stems of 1-year-old olive plants 90 days after inoculation with *P. savastanoi* pv. savastanoi NCPPB 3335 (wild type), Psv48ΔA (cured of pPsv48A) or Psv48ΔAB (cured of pPsv48A and pPsv48B). (**B**) Images of knots induced by the indicated GFP-tagged *P. savastanoi* pv. savastanoi strains on young micropropagated olive plants. (**C**) Complementary epifluorescence microscopy images of knots induced by the indicated strains.

*P. savastanoi* pv. savastanoi strains NCPPB 3335, Psv48ΔA (cured of pPsv48A), and Psv48ΔAB (cured of both pPsv48A and pPsv48B) were inoculated on the stem of 1 year-old olive plants. In agreement with previous reports [13], [41], [42], the wild-type strain induced typical dark brown hyperplastic knots on the stems of the olive plants at 90 days post-inoculation (dpi), whereas symptoms induced by cured strains Psv48ΔA and Psv48ΔAB were less severe (Figure 3A), probably due to the lack of the pPsv48A-encoded *ptz* gene. No visible symptoms were observed in the stems of control plants inoculated with a solution of MgCl_2_ (not shown).

*P. savastanoi* pv. savastanoi strains NCPPB 3335, Psv48ΔA and Psv48ΔAB were tagged with the green-fluorescent protein (GFP) using plasmid pLRM1-GFP [14] and inoculated at a cell concentration of approximately 10^3^ cfu on the stem of young micropropagated olive plants. In agreement with data reported by Rodríguez-Moreno *et al.* [14], strain NCPPB 3335 induced swelling of the stem tissue already observed at 7 dpi. As the swollen tissues continued to grow, typical hyperplastic knots were clearly visible at 28 dpi. In contrast, symptoms induced by the plasmid-cured derivatives were less severe. In all cases, swelling of the tissue evolved into attenuated hyperplastic knots, also showing a slight necrosis at 28 dpi (Figure 3B). Growth and survival of the different strains in the olive tissue was tested for all three strains. As reported for the wild-type strain [14], Psv48ΔA and Psv48ΔAB were able to multiply in the olive tissue during the first week post-inoculation reaching around 10^7^–10^8^ cfu per knot at 7 dpi (not shown). No significant difference in the number of cfu extracted from the olive plants was observed at 30 dpi between the wild type strain and any of the mutants tested. In all cases, the total number of cfu extracted per knot was about 10^8^.

As we previously reported [14], we were able to monitor *P. savastanoi* pv. savastanoi infection in real time using epifluorescence microscopy in plants infected with GFP-tagged strains. Despite the reduced knot size observed in plants infected with plasmid-cured strains, knots induced by the wild-type strain, Psv48ΔA and Psv48ΔAB exhibited a similar pattern of fluorescence emission composed by green fluorescent clusters that spanned the entire surface of the knot at 28 dpi (Figure 3C). The localization of GFP-tagged bacterial cells in knot tissues was monitored by epifluorescence and scanning confocal laser microscopy. As it occurs with the wild-type strain [14], transverse sections of knots induced by Psv48ΔA and Psv48ΔAB at 28 dpi clearly showed expanded areas of green fluorescent spots colonizing the apoplast as well as the internal open cavities and periphery of the knot tissues (not shown). Together, and in agreement with a previous report [41], all these results suggest that plasmid-cured *P. savastanoi* pv. savastanoi strains are able to multiply, survive and invade olive tissue as efficiently as the wild-type strain.

**Figure 4:**
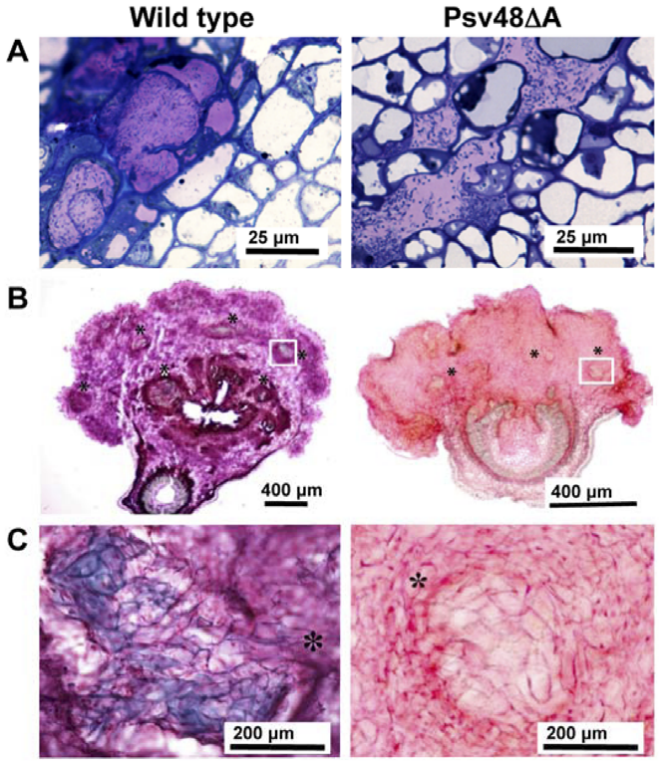
Microscopic analysis of knots. Young micropropagated olive plants were inoculated with *P. savastanoi pv. savastanoi* NCPPB 3335 (wild type) and Psv48ΔA (cured of pPsv48A). (**A**) Light microscopy images of semithin cross sections of knots (35 dpi) stained with toluidine (B, C) Cross-sections of knots, collected at 28 dpi, stained with methylene blue-picrofuchsin. Asterisks indicate the position of newly formed xylem vessels. (**B**) Parenchymatous-like cells showing a blue-purple stain of the cell walls (wild type) due to the formation of secondary walls during differentiation. (**C**) Detail of newly formed bundles of xylem vessels.

To view the parenchymal tissues of the olive plant knots in more detail, transverse semi-thin sections of the knots induced at 35 dpi by NCPPB 3335-GFP and Psv48ΔA were stained with toluidine blue and visualized by light microscopy. The characteristic internal cavities filled by bacteria formed in knots induced by the wild-type strain [15] were also visualized in knots induced by Psv48ΔA (Figure 4A). In addition, transverse sections of knots induced at 28 dpi by the wild-type strain stained with methylene blue-picrofuchsin showed newly formed bundles of spiral xylem vessels stained in purple-blue (secondary cell wall) inside the hypertrophied area (Figure 4B). In contrast, the smaller size of the tumors induced by Psv48ΔA was consistent with a lower presence of spiral vessels whose cells were not blue-stained, indicating that they were not completely differentiated into xylem cells containing secondary cell walls (Figure 4C). Thus, the development of mature xylem vessels within the knots induced by *P. savastanoi* pv. savastanoi NCPPB 3335 on young micropropagated olive plants seems to be partially dependent on the gene content of plasmid pPsv48A.

## Discussion

Plasmids are considered the predominant factors mediating horizontal gene transfer between bacteria in the environment [43]. Likewise, plasmids were shown to be very important vehicles for the dissemination of genes with agricultural value in the *P. syringae* group and other bacterial plant pathogens [24], [25], [44]. In the genomics era, the availability of closed plasmid sequences is pivotal to understand how plasmids originate, their gene dynamics and their role in gene trading in the bacterial community. The recent advances in sequencing technologies and reductions in cost have yielded a very large amount of nucleotide sequences in very little time, and resulted in a consequent shift towards the generation of an increasing amount of draft genome sequences [45], [46], [47]. This imposes a range of difficulties to make full use of genome data [46], especially with microbial genomes, and provides a very fragmented set of plasmid sequences, because these molecules usually contain a large amount of repeated sequences that make assembly difficult [9], [48]. Indeed, although the genomes of 39 strains of the *P. syringae* group will soon be sequenced, closed plasmid sequences are available for only four of these strains (see http://pseudomonas-syringae.org/). Here we present the closed sequence of the native plasmid complement of *P. savastanoi* pv. *savastanoi* strain NCPPB 3335, whose draft genome is available [16], and demonstrate that at least one of these plasmids is required for full virulence in olive plants.

Sequencing of the plasmids was greatly hampered by the large amount of repeated sequences they contain and share among them, and that are common in plasmids of the *P. syringae* group [17], [21], [49]. For instance, pPsv48C contains two copies of IS*51* that show 1 nt difference over 1,312 nt and two identical copies of IS*Psy16* (1,461 nt), which recombine between them resulting in an active flipping of the intervening DNA (Figure 2). Additionally, plasmids B and C share an estimated 25% of their sequences, often showing a high degree of identity. An extreme case was pPsv48A containing three copies of a large DNA region, encompassing a putative effector (PSPSV_A0005, PSPSV_A0035 and PSPSV_A0046) and associated adjacent DNA, that showed areas of up to 5.9 kb with 95% nucleotide identity. These repetitions resulted in the misassembly of the pyrosequencing data, which we solved here by cloning and sequencing a collection of EcoRI fragments obtained, when possible, from individualized plasmids and by sequencing of PCR products for gap closure. The misassembly of the draft genome of strain NCPPB 3335 is illustrated by its comparison with the assembled, curated sequence of pPsv48B. Although this plasmid contains a very low number of mobile elements, it was distributed among four contigs in the pyrosequencing data (counting only those with at least 1 kb of continuous homology with pPsv48B with >99% identity; contigs ADMI01000061 to ADMI01000064). A 3 kb pair-end library analysis significantly improved assembly and resulted in a single supercontig for pPsv48B (ASAPContig021; 51,830 nt), but there were still near 4.6 kb missing and 15 mismatches in the pair-end assembly as well as extra DNA that did not belong to this plasmid (Figure S5). A comparison of the draft genome with plasmids pPsv48A and pPsv48C indicate that they are distributed among a much larger number of contigs (not shown), many of which contain repeated sequences. Likewise, there are inconsistencies in between the draft genome and the closed plasmid sequence, with some of the plasmid sequences missing from the draft genome; for example, only one homolog of gene PSPPH_1525 is found in the draft genome, although we confirmed the existence of three copies (PSPSV_A0005, PSPSV_A0035 and PSPSV_A0046) in plasmid pPsv48A by PCR, sequencing and DNA hybridization. Therefore, future analyses of plasmid population genetics in *P. syringae* would require the generation of genomic sequences of sufficient quality to guarantee the closure.

There is a large variability in the coding percentage for each plasmid, which is partially correlated with the content in putative mobile elements (Table 1), that amount to 24.8 to 29.5% of pPsv48A and pPsv48C, respectively, but only a 3.7% of pPsv48B. These percentages are well out of the usual ranges, which average 5–15% for plasmids larger than 20 kb [48], and might indicate a high level of transposition and recombination in these native plasmids.

The phylogenetic analysis of the *repA* gene (Figure S3) strongly suggests that plasmids pPsv48B and pPsv48C originated by duplication of an ancestral plasmid; their *repA* deduced products show nearly 98% aa identity, with seven nonsynonymous substitutions of which four are located within the first 20 amino acids. This is in contrast with previous observations of a higher variability in the C-terminal end of RepA proteins of PFP plasmids [18], [20], and suggests that modifications in the N-terminal end might be important to avoid incompatibility in co-resident PFP plasmids. Although pPsv48C contains a second putative replication protein gene (*repL*), we were unable to demonstrate its functionality in *E. coli* and diverse pseudomonads.

Two of the putative virulence genes found in the plasmids code for putative effector genes (PSPSV_A0028, *hopAF1*, and PSPSV_B0010, *hopAO1*) homologous to effectors already found in bacteria of the *P. syringae* complex [16] and that are preceded by typical *hrp* promoters, suggesting that they might be part of the HrpL regulon in strain NCPPB 3335. The availability of the complete, closed plasmid sequences allowed us to establish that they contain only two of the known effectors, as opposed to our previous results suggesting the presence of effectors *hopD1* and *hopW1* in the plasmids of NCPPB 3335 [16], [21]. Effector gene *hopAO1*, located in pPsv48B, is 87% identical to the one present in *P. syringae* pv. *tomato* DC3000 [34], and codes for a putative tyrosine phosphatase. Gene *hopAF1*, harbored by pPsv48A, is widely distributed in the *P. syringae* complex, and is unusual in that in strain NCPPB 3335 it is included into a transposon, which might facilitate its dissemination. Although this type of mobile element is not common in the *P. syringae* complex, a functional transposon containing effector gene *avrPphE* (syn. *hopX1*) has been described in *P. syringae* pv. tomato DC3000 [50].

Plasmid pPsv48A contains three alleles (PSPSV_A0005, PSPSV_A0035 and PSPSV_A0046) of a hypothetical gene widely conserved among plant-associated proteobacteria that are preceded by a *hrp* box. The closest homologue, PSPPH_1525, from *P. syringae* pv. phaseolicola 1448A, was shown to be inducible by HrpL and suspected to be a T3SS substrate, although secretion could not be shown due to the large size of the protein [34]. Additionally, homolog *mlr6361*, from *Mesorhizobium loti*, is responsible for restriction of host range in *Lotus halophilus*, and the T3SS-dependent translocation of its product was unequivocally demonstrated [51], [52]. Together, these data suggest that these large genes might code for T3SS effectors. Although plasmid curing did not reveal any apparent role for these three loci (PSPSV_A0005, PSPSV_A0035 and PSPSV_A0046), two lines of evidence support the idea that they are functional and relevant for the bacterial life cycle. First is the fact that they are highly conserved among a wide range of plant-related bacteria belonging to very different phylogenetic taxa, such as *Bradyrhizobium* and *Ralstonia*, suggesting that they are involved in basic processes of the interaction with the plant hosts. Secondly, they are very large CDSs, from 7.1 to 7.8 kb, and are located in a plasmid, pPsv48A, that contains mobile elements accounting for nearly a quarter of its size; in spite of that, the CDSs do not contain any premature stops or any insertion of a mobile element, suggesting that they contribute to increasing fitness. Remarkably, these CDSs contain a variable number of tandem repeats of around 126 nt that conform an Armadillo-like domain (InterPro IPRO11989 and IPR016024); the superhelical structure of this domain is suited to binding large substrates, such as proteins and nucleic acids. In line with this, the products of *mlr6361* and *mlr6331*, both homologs of PSPPH_1525, interacted between them in a yeast-two hybrid assay [53]. It is conceivable that variations in the number and type of repeat could afford specificity during the interaction with the plant host, as it happens with type III effectors of the TAL family of *Xanthomonas* [54] and, indeed, a rapid loss/gain of repeats has occurred frequently during evolution in this family of proteins.

Genes for phytohormone biosynthesis have a disparate genomic localization in different tumor-inducing strains of *P. savastanoi*, with genes for the biosynthesis of cytokinins preferentially located in plasmids of the pPT23A-family in *P. savastanoi* pv. savastanoi [21], [26], [28], [55]. In accordance with our previous macroarray hybridization results [21], we found gene *ptz* in pPsv48A. This gene is well conserved among an exceptionally wide panoply of bacteria with very diverse lifestyles, ranging from enterobacteria to free living cyanobacteria, although it is also present in various plant pathogens. These in particular are representative of different pathogenic strategies, including pathogens that induce tumors, such as *P. savastanoi* pv. savastanoi and *Agrobacterium* spp., or colonize the vascular system, such as *X. albilineans* and *Ralstonia solanacearum*, although the role of cytokinins in most of these pathosystems is as yet unknown. Gene *ptz* is included in a potential genomic island in pPsv48A, characterized by a low G+C content, although blast comparisons did not reveal the presence of the complete island in any other bacterium; additionally, we did not find any terminal repeated sequence typical of genomic islands, raising the possibility that it could be an artifact. Symptoms induced in olive plants by Psv48ΔA (Figure 3), which lacks the *ptz* gene, are in agreement with data previously reported by Iacobellis and co-workers [41]. In fact, symptoms induced by this plasmid-cured derivative nearly resembled those induced in 1-year-old olive and oleander shoots by a *P. savastanoi* pv. nerii strain cured of a pCK plasmid which encodes *ptz*. Growth and survival of this cytokinin-deficient strain in young micropropagated olive plants was shown to be similar to that of the wild type strain [14]. Cytokinins are involved in the regulation of procambial cell differentiation into vascular cells [56]. Thus, the formation of immature xylem vessels observed in tumors induced by Psv48ΔA (Figure 4) is most likely a consequence of the lack of the *ptz* gene in this strain which could result in a deficiency in cytokinin biosynthesis. Symptoms induced by Psv48ΔAB were similar to those induced by Psv48ΔA (Figure 3). This observation indicates that the visible effect on virulence of pPsv48A could be dominant over that of pPsv48B. However, we could not test this hypothesis, since plasmid-cured derivatives lacking only pPsv48B or pPsv48C could not be constructed using Tn*5*-GDYN1.

In summary, we report the complete sequence and annotation of three native plasmids from P. savastanoi pv. savastanoi NCPPB 3335, and demonstration of a link between pPsv48A and virulence. Additional functional analysis of specific plasmid-encoded genes in NCPPB 3335 will help us to uncover the precise role of each of these three plasmids in the virulence and host range of *P. savastanoi* pv. savastanoi.

## Materials and Methods

### Bacterial strains and growing conditions

The bacterial strains and plasmids used in this study are listed in Table S6. *Pseudomonas* spp. and *Escherichia coli* strains were grown in LB medium [57] at 28 and 37°C respectively. GFP-tagged derivatives of *P. savastanoi* pv. savastanoi NCPPB 3335, Psv48ΔA and Psv48ΔAB harboring the pLRM1-GFP plasmid (Table S6) are referred to here as NCPPB 3335-GFP, Psv48ΔA-GFP and Psv48ΔAB-GFP, respectively. Transformation of electrocompetent *P. savastanoi* pv. savastanoi cells with pLRM1-GFP was performed as previously described [13]. When necessary, media were supplemented with (final concentrations in µg/ml): ampicillin, 100; kanamycin, 7, to select for Tn*5*-GDYN1, or 50, in the remaining cases; nitrofurantoin, 100; 5-bromo-4-chloro-3-indolyl-beta-D-galactopyranoside (X-Gal), 40; and isopropyl-beta-D-thiogalactopyranoside (IPTG), 0.5 mM.

### Molecular techniques

For sequencing, we attempted to individualize and separately purify each of the native plasmids of strain NCPPB 3335. Plasmid pPsv48C and pPsv48A::Tn*5*-GDYN1 were successfully isolated from strains Psv48ΔAB and B728a(pPsv48A::Tn*5*-GDYN1), respectively (see Table S6). We used strain Psv48ΔA as a source of pPsv48B, from which a mixture of pPsv48B and pPsv48C was obtained. We extracted native plasmid DNA using an alkaline lysis method [21], [58] and further purified the plasmids by isopycnic centrifugation in CsCl [59]. Intact plasmids were separated by electrophoresis in 0.8% agarose gels using 1× TAE [13], [19]. Transposon mutagenesis and plasmid curing was carried out essentially as described by Brom *et al.* [31], except that derivatives of strain NCPPB 3335 containing the transposon were selected on LB containing kanamycin and nitrofurantoin, that insertions in plasmids were identified by their change in mobility after electrophoresis in agarose gels and that plasmid cured derivatives were selected in media containing 5% sucrose.

To assay the ability of gene *repL* (PSPSV_C0043) to sustain autonomous replication, appropriate PCR products containing the complete CDS and 240 nt upstream of the start codon were cloned in the vectors pSW25T and pSW29T, which contain an R6K origin of replication [60]. In the same way, we constructed clones that also contained the downstream CDS (PSPSV_C0044), coding for a putative entry exclusion protein. All of the cloned fragments were identical to the original sequence, as determined by DNA sequencing. The replication ability of the resulting recombinant plasmids was tested by transformation into *E. coli* DH5α, *P. syringae* pv. syringae B728a, *P. syringae* pv. phaseolicola 1448A and *P. savastanoi* pv. savastanoi NCPPB 3335. pAori1, containing *repA* from a *P. syringae* pv. tomato PT23 native plasmid [17], was used as a replication control.

PCR reactions, using a *Taq* polymerase (BioTaq, Bioline, London, UK) or a high fidelity enzyme blend (Expand High-Fidelity, Roche), restriction enzyme digestions, cloning, transformation of constructs and minipreparation of *E. coli* plasmids were all conducted using standard methodology [57]. When needed, PCR products were cloned using either pGEM-T Easy Vector System I kit (Promega, Corp, Madison, WI) or pCR2.1 (Invitrogen). Oligonucleotide primers were designed using Primer3plus software [61]. All DNA sequencing was done at Macrogen Inc. (Seoul, Korea).

### Plasmid sequencing and assembly

For the sequence assembly we used two sets of sequences, comprising those of the draft genome dataset of strain NCPPB 3335 and cloned EcoRI fragments from individual plasmids (see below). The draft genome was obtained by 454 pyrosequencing at 15× depth of total DNA from strain NCPPB 3335 and it was composed of 287 contigs larger than 1.5 kb [accession no. NZ_ADMI00000000; 16]. For sequencing of the cloned EcoRI fragments, DNA from each purified native plasmid was digested with EcoRI and the resulting fragments were ligated en masse into the *E. coli* vectors pBluescript SK II (Stratagene, La Jolla, CA) or pGEM-3Z (Promega Corp, Madison, WI). Constructs were then transformed into *E. coli* DH5α or XL1-Blue cells, and recombinant plasmids were digested with EcoRI and separated by gel electrophoresis, along with native plasmid DNA digested with the same enzyme. We only end sequenced those constructs with a single EcoRI insert that co-migrated with a band present in the native plasmid restriction profile. These sequences were compared to the draft genome dataset using the Blast algorithm included in BioEdit Sequence Alignment Editor (Ibis Therapeutics, Carlsbad, CA, USA) in order to identify those contigs containing plasmid DNA. Confirmation of the order and orientation of EcoRI fragments in contigs, as well as the bridging of contigs into a circular structure, was done by PCR amplification and sequencing of at least 0.5 kb on each side of the DNA surrounding the junction of two consecutive EcoRI fragments, as well as by sequencing of selected complete EcoRI fragments.

DNA sequences were manipulated, assembled and annotated using the Vector NTI suite (Informax, Inc., Frederick, MD) and Artemis [62], [63]. Annotation was done using Blast2GO [64] and RAST [65] and it was manually refined using BLAST outputs [66]. Pairwise alignments between the assembled plasmids was made with WebACT [67] and viewed using ACT [68]. IS elements and their borders were identified, by BLAST comparison, using the IS Finder database (http://www-is.biotoul.fr). DNA or amino acid sequences alignment using Clustal and construction of phylogenies was done with MEGA5.02 [69]. Trees were constructed using the Neighbor-Joining and Maximum Parsimony methods, and the option pairwise deletion was chosen to eliminate position with gaps; confidence levels of the branching points were determined using 2,000 bootstrap replicates. The presence of genomic islands was predicted using the web site IslandViewer (http://www.pathogenomics.sfu.ca/islandviewer) [70], which uses three methods for island prediction.

Plasmid sequences were deposited in EMBL databases under accession numbers FR820585 (pPsv48A), FR820586 (pPsv48B) and FR820587 (pPsv48C).

### Plant inoculation and growing conditions

Olive plants (*Olea europaea* L.) derived from seeds germinated *in vitro* (originally collected from a cv. Arbequina plant) were micropropagated and rooted, as previously described [14], in Driver Kuniyuki Walnut (DKW) medium [71]. Rooted explants were transferred to DKW medium without hormones and kept for at least two weeks in a growth chamber at 25±1°C with a 16-h photoperiod prior to infection. The olive plants used for *in vitro* studies were 60 to 80-mm long (stem diameter 1 to 2 mm) and contained three to five internodal fragments.

Micropropagated olive plants were wounded by excision of an intermediate leaf and infected in the stem wound with a bacterial suspension under sterile conditions. For this purpose, bacterial lawns were grown for 48 h in LB plates and resuspended in 10 mM MgCl_2_. Bacterial suspensions were adjusted to an OD_600_ of 0.1, corresponding to 10^7^ colony forming units (cfu)/ml, and 2 µl (approximately 10^4^ cfu) were used to infect plant wounds; plants were then incubated in a growth chamber at 25±1°C with a 16-h photoperiod and a light intensity of 35 µmoles×m^2^/s. To estimate population dynamics, we prepared macerates from the infected explants at different time points that were spotted onto LB plates to recover and count bacteria as previously described [14]. Population densities were averaged from at least three replicates. The morphology of the olive plants infected with bacteria was visualized using a stereoscopic microscope (Leica MZ FLIII).

To analyze the pathogenicity of *P. savastanoi* pv. savastanoi isolates in one-year-old olive explants, micropropagated olive plants were transferred to soil and maintained in a greenhouse at 27°C with a relative humidity of 58% under natural daylight. The plants were wounded at five sites on the main stem. The wounds, which were 0.5 cm deep and spanned from the stem surface to the cambial area, were made with a sterile scalpel and were infected with approximately 10^6^ cfu of the strain being tested using bacterial suspensions prepared as previously described [12], [13]. Morphological changes, scored 90 days after infection, were captured with a high-resolution digital camera (Nikon DXM 1200), and the images were processed using Adobe Photoshop CS software.

### Real-time monitoring of bacterial infection by epifluorescence microscopy

To visualize bacterial infection within tumors in real time, whole knots were directly examined with a stereoscopic fluorescence microscope (Leica MZ FLIII) equipped with a 100-W mercury lamp and a GFP2 filter (excitation, 480/40 nm). Images were captured using a high-resolution digital camera (Nikon DXM 1200), and the images were processed using Adobe Photoshop CS software.

To visualize bacterial infection within the tumors of the olive plants, the knots were sampled on different dpi at locations 1 cm above and 1 cm below the inoculation point. These samples were fixed and embedded in agarose as previously described [15]. Samples were fixed overnight at 4°C in 2.5% paraformaldehyde (PFA) prepared in 0.1 M phosphate buffer, pH 7.4. The fixed samples were then transferred into 2.5% PFA with an ascending gradient of 10%, 20%, and 30% sucrose for 10, 20, and 30 min, respectively. Finally, samples were embedded in 7% low-melting-point agarose and cooled to 4°C. Sections (40 and 60 µm thick) were cut from the knot samples using a freezing microtome (Leica CM1325). Fluorescence of the bacterial cells within knot sections was visualized by epifluorescence microscopy using a Nikon Microphot FXA microscope.

### Toluidine blue and methylene blue-picrofuchsin stains

Olive knot samples, sectioned and fixed as described above, were stained for 10 s in 1% methylene blue. Then they were washed in ethanol (96%), followed by distilled water and finally stained for 5 min in picrofuchsin. Picrofuchsin contained 0.1% acid fuchsin in a saturated picric acid solution. Semithin (1-µm-thick) sections of the knots were cut using an ultramicrotome (Ultracut E; Leica, Germany), mounted on glass slides and stained with 1% toluidine blue. Stained sections were dehydrated, mounted on slides with Canadian balsam and visualized with a Nikon Eclipse 800 light microscope.

## Supporting Information

Figure S1 **Structure of the effector transposon IS** ***Psy30*** **found in pPsv48A.** Open reading frames are indicated by block arrows, terminal inverted repeats as red rectangles, and *hrp* boxes as black triangles. Grey bars indicate collinear regions, with the percentage of identity shown. IS*Psy30* was compared to the genome of *P. syringae* pv. tomato DC3000 (accession no. AE016853) and the larger plasmid from *P. syringae* pv. phaseolicola 1448A (accession no. CP000059).
(PPT)

Figure S2 **Inverted repeats of MITE** ***Psy3*** **.** Comparison of the repeats of MITE*Psy3* and the Tn*3* family transposon IS*Thsp9*, from *Thiomonas* sp. Identical nt in at least three sequences are boxed in black.
(PPT)

Figure S3 **Phylogenetic analysis of full nucleotide sequences of the** ***repA*** **gene from PFP plasmids from strains of the** ***P. syringae*** **complex.** The evolutionary history was inferred by Neighbor-Joining using MEGA5 [69]; evolutionary distances were computed using the Maximum Composite Likelihood method, and pairwise deletion, and are in the units of the number of base substitutions per site; a similar topology was obtained using Maximum parsimony with default settings. The percentages of replicate trees in which the associated taxa clustered together in the bootstrap test (2000 replicates) are shown next to the branches. The tree was constructed with 44 *repA* sequences previously described [20], plus those from the three plasmids of *P. savastanoi* pv. savastanoi NCPPB 3335 (arrows) and using the *repA* from *Thiomonas intermedia* K12 plasmid pTINT01 (accession no. CP002022, locus tag Tint_3234) as an outgroup; the pathovar of origin of each sequence is shown after the name of the plasmid. Phylogenetic groups are as described [20]; groups A, B and D are shown as triangles proportional to the number of sequences they contain; numbers after the name of groups indicate the genomospecies of the pathovars from which the plasmids were isolated.
(PPT)

Figure S4 **Conservation of plasmids backbone.** Pairwise blast alignment of native plasmids pPMA4326A (AY603979; top), pPsv48B (middle) and pPsv48C (bottom), done with WebACT and visualized with ACT; red and blue indicate collinear and inverted regions of identity, respectively.. Only those matches longer than 100 nt with at least 80% identity are shown.
(PPT)

Figure S5 **Example of inadequate assembly of plasmid sequences in the draft genome of** ***P. savastanoi*** **pv. savastanoi NCPPB 3335.** Comparison of the closed, curated sequence of pPsv48B (upper sequence; 45,220 nt) with supercontig ASAPContig021 (lower sequence; 51,830 nt; https://asap.ahabs.wisc.edu/asap/home.php) obtained after 454 shotgun sequencing and pair-end library analysis. A Blastn comparison was done with WebACT and visualized with ACT; red and blue indicate collinear and inverted regions of identity, respectively.
(PPT)

Table S1 **Number of putative genes predicted in the annotation of the native plasmids of** ***P. savastanoi*** **pv.** ***savastanoi*** **NCPPB 3335, separated by functional categories.**
(DOC)

Table S2 **Plasmid features with low (<50%) or high (>62%) G+C content.**
(DOC)

Table S3 **Genes coding for components of Type IV secretion systems.**
(DOC)

Table S4 **Putative virulence genes found in the native plasmids from** ***P. savastanoi*** **pv. savastanoi NCPPB 3335.**
(DOC)

Table S5 **Type and number of mobile elements found in the native plasmids of** ***P. savastanoi*** **pv. savastanoi NCPPB 3335.**
(DOC)

Table S6 **Bacterial strains and plasmids used in this work.**
(DOC)

## References

[1] Smith IM, Dunez J, Lelliott RA, Phillips DH, Archer SA (1988). European handbook of plant diseases.

[2] Gardan L, Shafik H, Belouin S, Broch R, Grimont F (1999). DNA relatedness among the pathovars of *Pseudomonas syringae* and description of *Pseudomonas tremae* sp. nov. and *Pseudomonas cannabina* sp. nov. (*ex* Sutic and Dowson 1959).. International Journal of Systematic Bacteriology.

[3] Young JM (2010). Taxonomy of *Pseudomonas syringae*.. Journal of Plant Pathology.

[4] Sarkar SF, Guttman DS (2004). Evolution of the core genome of *Pseudomonas syringae*, a highly clonal, endemic plant pathogen.. Applied and Environmental Microbiology.

[5] Qi M, Wang D, Bradley CA, Zhao Y (2011). Genome sequence analyses of *Pseudomonas savastanoi* pv. *glycinea* and subtractive hybridization-based comparative genomics with nine Pseudomonads.. PLoS ONE.

[6] Green S, Studholme DJ, Laue BE, Dorati F, Lovell H (2010). Comparative genome analysis provides insights into the evolution and adaptation of *Pseudomonas syringae* pv. *aesculi* on *Aesculus hippocastanum*.. PLoS ONE.

[7] Joardar V, Lindeberg M, Jackson RW, Selengut J, Dodson R (2005). Whole-genome sequence analysis of *Pseudomonas syringae* pv. phaseolicola 1448A reveals divergence among pathovars in genes involved in virulence and transposition.. Journal of Bacteriology.

[8] Studholme DJ, Ibanez SG, MacLean D, Dangl JL, Chang JH (2009). A draft genome sequence and functional screen reveals the repertoire of type III secreted proteins of *Pseudomonas syringae* pathovar *tabaci* 11528.. BMC Genomics.

[9] Baltrus DA, Nishimura MT, Romanchuk A, Chang JH, Mukhtar MS (2011). Dynamic evolution of pathogenicity revealed by sequencing and comparative genomics of 19 *Pseudomonas syringae* isolates.. PLoS Pathogens.

[10] Iacobellis NS, Maloy OC, Murray TD (2001). Olive knot.. Encyclopedia of Plant Pathology.

[11] Quesada JM, Penyalver R, Pérez-Panadés J, Salcedo CI, Carbonell EA (2010). Dissemination of *Pseudomonas savastanoi* pv. *savastanoi* populations and subsequent appearance of olive knot disease.. Plant Pathology.

[12] Penyalver R, Garcia A, Ferrer A, Bertolini E, Quesada JM (2006). Factors affecting *Pseudomonas savastanoi* pv. *savastanoi* plant inoculations and their use for evaluation of olive cultivar susceptibility.. Phytopathology.

[13] Pérez-Martínez I, Rodríguez-Moreno L, Matas IM, Ramos C (2007). Strain selection and improvement of gene transfer for genetic manipulation of *Pseudomonas savastanoi* isolated from olive knots.. Research in Microbiology.

[14] Rodríguez-Moreno L, Barceló-Muñoz A, Ramos C (2008). *In vitro* analysis of the interaction of *Pseudomonas savastanoi* pvs. *savastanoi* and *nerii* with micropropagated olive plants.. Phytopathology.

[15] Rodríguez-Moreno L, Jiménez AJ, Ramos C (2009). Endopathogenic lifestyle of *Pseudomonas savastanoi* pv. *savastanoi* in olive knots.. Microbial Biotechnology.

[16] Rodríguez-Palenzuela P, Matas I, Murillo J, López-Solanilla E, Bardaji L (2010). Annotation and overview of the *Pseudomonas savastanoi* pv. savastanoi NCPPB 3335 draft genome reveals the virulence gene complement of a tumour-inducing pathogen of woody hosts.. Environmental Microbiology.

[17] Murillo J, Keen NT (1994). Two native plasmids of *Pseudomonas syringae* pathovar tomato strain PT23 share a large amount of repeated DNA, including replication sequences.. Molecular Microbiology.

[18] Gibbon MJ, Sesma A, Canal A, Wood JR, Hidalgo E (1999). Replication regions from plant-pathogenic *Pseudomonas syringae* plasmids are similar to ColE2-related replicons.. Microbiology.

[19] Sesma A, Sundin GW, Murillo J (1998). Closely related replicons coexisting in the phytopathogen *Pseudomonas syringae* show a mosaic organization of the replication region and altered incompatibility behavior.. Applied and Environmental Microbiology.

[20] Ma Z, Smith JJ, Zhao Y, Jackson RW, Arnold DL (2007). Phylogenetic analysis of the pPT23A plasmid family of *Pseudomonas syringae*.. Applied and Environmental Microbiology.

[21] Pérez-Martínez I, Zhao Y, Murillo J, Sundin GW, Ramos C (2008). Global genomic analysis of *Pseudomonas savastanoi* pv. savastanoi plasmids.. Journal of Bacteriology.

[22] Sundin GW, Jacobs JL (1999). Ultraviolet radiation (UVR) sensitivity analysis and UVR survival strategies of a bacterial community from the phyllosphere of field-grown peanut (*Arachis hypogeae* L.).. Microbial Ecology.

[23] Zhao YF, Ma ZH, Sundin GW (2005). Comparative genomic analysis of the pPT23A plasmid family of *Pseudomonas syringae*.. Journal of Bacteriology.

[24] Sundin GW (2007). Genomic insights into the contribution of phytopathogenic bacterial plasmids to the evolutionary history of their hosts.. Annual Review of Phytopathology.

[25] Sundin GW, Murillo J, Jackson RW (2009). Gene traders: characteristics of native plasmids from plant pathogenic bacteria.. Plant pathogenic bacteria: genomics and molecular biology.

[26] Macdonald EMS, Powell GK, Regier DA, Glass NL, Roberto F (1986). Secretion of zeatin, ribosylzeatin, and ribosyl-1″-methylzeatin by *Pseudomonas savastanoi*: plasmid-coded cytokinin biosynthesis.. Plant Physiology.

[27] Glass NL, Kosuge T (1988). Role of indoleacetic acid lysine synthetase in regulation of indoleacetic-acid pool size and virulence of *Pseudomonas syringae* subsp. *savastanoi*.. Journal of Bacteriology.

[28] Silverstone SE, Gilchrist DG, Bostock RM, Kosuge T (1993). The 73-kb pIAA plasmid increases competitive fitness of *Pseudomonas syringae* subspecies *savastanoi* in oleander.. Canadian Journal of Microbiology.

[29] Vivian A, Murillo J, Jackson RW (2001). The role of plasmids in phytopathogenic bacteria: mobile arsenals?. Microbiology.

[30] Murillo J, Butcher D, Jackson RW, Sundin GW, Vivian A, Iacobellis NS, Collmer A, Hutcheson S, Mansfield JW, Morris CE (2003). Methods for the identification of virulence genes in *Pseudomonas syringae*.. *Pseudomonas syringae* and related pathogens Biology and genetics.

[31] Brom S, García de los Santos A, Stepkowsky T, Flores M, Dávila G (1992). Different plasmids of *Rhizobium leguminosarum* bv. phaseoli are required for optimal symbiotic performance.. Journal of Bacteriology.

[32] Flores M, Brom S, Stepkowski T, Girard ML, Dávila G (1993). Gene amplification in *Rhizobium*: identification and *in vivo* cloning of discrete amplifiable DNA regions (amplicons) from *Rhizobium leguminosarum* biovar *phaseoli*.. Proceedings of the National Academy of Science USA.

[33] Underwood W, Zhang S, He SY (2007). The *Pseudomonas syringae* type III effector tyrosine phosphatase HopAO1 suppresses innate immunity in *Arabidopsis thaliana*.. The Plant Journal.

[34] Vencato M, Tian F, Alfano JR, Buell CR, Cartinhour S (2006). Bioinformatics-enabled identification of the HrpL regulon and type III secretion system effector proteins of *Pseudomonas syringae* pv. phaseolicola 1448A.. Molecular Plant-Microbe Interactions.

[35] Stavrinides J, Ma W, Guttman DS (2006). Terminal reassortment drives the quantum evolution of type III effectors in bacterial pathogens.. PLoS Pathogens.

[36] Stevens C, Bennett MA, Athanassopoulos E, Tsiamis G, Taylor JD (1998). Sequence variations in alleles of the avirulence gene *avrPphE.R2* from *Pseudomonas syringae* pv. *phaseolicola* lead to loss of recognition of the AvrPphE protein within bean cells and a gain in cultivar-specific virulence.. Molecular Microbiology.

[37] Bardaji L, Añorga M, Jackson RW, Martínez-Bilbao A, Yanguas N (2011). Miniature transposable sequences are frequently mobilized in the bacterial plant pathogen *Pseudomonas syringae* pv. phaseolicola.. PLoS *ONE*.

[38] Schlüter A, Krahn I, Kollin F, Bönemann G, Stiens M (2007). IncP-1{beta} plasmid pGNB1 isolated from a bacterial community from a wastewater treatment plant mediates decolorization of triphenylmethane dyes.. Applied and Environmental Microbiology.

[39] Sesma A, Sundin GW, Murillo J (2000). Phylogeny of the replication regions of pPT23A-like plasmids from *Pseudomonas syringae*.. Microbiology.

[40] Krid S, Rhouma A, Quesada JM, Penyalver R, Gargouri A (2009). Delineation of *Pseudomonas savastanoi* pv. savastanoi strains isolated in Tunisia by random-amplified polymorphic DNA analysis.. Journal of Applied Microbiology.

[41] Iacobellis NS, Sisto A, Surico G, Evidente A, DiMaio E (1994). Pathogenicity of *Pseudomonas syringae* subsp. *savastanoi* mutants defective in phytohormone production.. Journal of Phytopathology.

[42] Pérez-Martínez I, Rodríguez-Moreno L, Lambertsen L, Matas IM, Murillo J (2010). Fate of a *Pseudomonas savastanoi* pv. savastanoi type III secretion system mutant in olive plants (*Olea europaea* L.).. Applied and Environmental Microbiology.

[43] Halary S, Leigh JW, Cheaib B, Lopez P, Bapteste E (2010). Network analyses structure genetic diversity in independent genetic worlds.. Proceedings of the National Academy of Sciences USA.

[44] Jackson RW, Vinatzer B, Arnold DL, Dorus S, Murillo J (2011). The influence of the accessory genome on bacterial pathogen evolution.. Mobile Genetic Elements.

[45] Nagarajan N, Cook C, Di Bonaventura M, Ge H, Richards A (2010). Finishing genomes with limited resources: Lessons from an ensemble of microbial genomes.. BMC Genomics.

[46] Fraser CM, Eisen JA, Nelson KE, Paulsen IT, Salzberg SL (2002). The value of complete microbial genome sequencing (you get what you pay for).. Journal of Bacteriology.

[47] Imelfort M, Edwards D (2009). De novo sequencing of plant genomes using second-generation technologies.. Briefings in Bioinformatics.

[48] Siguier P, Filee J, Chandler M (2006). Insertion sequences in prokaryotic genomes.. Current Opinion in Microbiology.

[49] Stavrinides J, Guttman D (2004). Nucleotide sequence and evolution of the five-plasmid complement of the phytopathogen *Pseudomonas syringae* pv. maculicola ES4326.. Journal of Bacteriology.

[50] Landgraf A, Weingart H, Tsiamis G, Boch J (2006). Different versions of *Pseudomonas syringae* pv. *tomato* DC3000 exist due to the activity of an effector transposon.. Molecular Plant Pathology.

[51] Sánchez C, Iannino F, Deakin WJ, Ugalde RA, Lepek VC (2009). Characterization of the *Mesorhizobium loti* MAFF303099 type-three protein secretion system.. Molecular Plant-Microbe Interactions.

[52] Okazaki S, Okabe S, Higashi M, Shimoda Y, Sato S (2010). Identification and functional analysis of Type III effector proteins in *Mesorhizobium loti*.. Molecular Plant-Microbe Interactions.

[53] Shimoda Y, Shinpo S, Kohara M, Nakamura Y, Tabata S (2008). A large scale analysis of protein-protein interactions in the nitrogen-fixing bacterium *Mesorhizobium loti*.. DNA Research.

[54] Boch J, Scholze H, Schornack S, Landgraf A, Hahn S (2009). Breaking the code of DNA binding specificity of TAL-Type III Effectors.. Science.

[55] Surico G, Iacobellis NS, Verma DPS (1992). Phytohormone and olive knot disease.. Molecular signals in plant-microbe interactions.

[56] Fukuda H (2004). Signals that control plant vascular cell differentiation.. Nature Reviews Molecular Cell Biology.

[57] Sambrook J, Fritsch EF, Maniatis T (1989). Molecular Cloning: a Laboratory Manual.

[58] Zhou C, Yang Y, Jong AY (1990). Miniprep in ten minutes.. BioTechniques.

[59] Murillo J, Shen H, Gerhold D, Sharma AK, Cooksey DA (1994). Characterization of pPT23B, the plasmid involved in syringolide production by *Pseudomonas syringae* pv. *tomato* PT23.. Plasmid.

[60] Demarre G, Guerout AM, Matsumoto-Mashimo C, Rowe-Magnus DA, Marliere P (2005). A new family of mobilizable suicide plasmids based on broad host range R388 plasmid (IncW) and RP4 plasmid (IncP-alpha) conjugative machineries and their cognate *Escherichia coli* host strains.. Research in Microbiology.

[61] Rozen S, Skaletsky HJ, Krawetz S, Misene S (2000). Primer3 on the WWW for general users and for biologist programmers.. Bioinformatics Methods and Protocols: Methods in Molecular Biology.

[62] Rutherford K, Parkhill J, Crook J, Horsnell T, Rice P (2000). Artemis: sequence visualisation and annotation.. Bioinformatics.

[63] Carver T, Berriman M, Tivey A, Patel C, Bohme U (2008). Artemis and ACT: viewing, annotating and comparing sequences stored in a relational database.. Bioinformatics.

[64] Conesa A, Gotz S, Garcia-Gomez JM, Terol J, Talon M (2005). Blast2GO: a universal tool for annotation, visualization and analysis in functional genomics research.. Bioinformatics.

[65] Aziz RK, Bartels D, Best AA, DeJongh M, Disz T (2008). The RAST server: Rapid annotations using subsystems technology.. BMC Genomics.

[66] Altschul SF, Madden TL, Schäffer AA, Zhang J, Zhang Z (1997). Gapped BLAST and PSI-BLAST: a new generation of protein database search programs.. Nucleic Acids Research.

[67] Abbott JC, Aanensen DM, Rutherford K, Butcher S, Spratt BG (2005). WebACT-an online companion for the Artemis Comparison Tool.. Bioinformatics.

[68] Carver TJ, Rutherford KM, Berriman M, Rajandream M-A, Barrell BG (2005). ACT: the Artemis Comparison Tool.. Bioinformatics.

[69] Tamura K, Peterson D, Peterson N, Stecher G, Nei M (2011). Molecular evolutionary genetics analysis using maximum likelihood, evolutionary distance, and maximum parsimony methods.. Molecular Biology and Evolution.

[70] Langille MGI, Brinkman FSL (2009). IslandViewer: an integrated interface for computational identification and visualization of genomic islands.. Bioinformatics.

[71] Driver JA, Kuniyuki AH (1984). *In vitro* propagation of Paradox walnut rootstock.. Hortscience.

