# Supplementary figures and images for: Sequence and Role in Virulence of the Three Plasmid Complement of the Model Tumor-Inducing Bacterium Pseudomonas savastanoi pv. savastanoi NCPPB 3335
Source: PLoS One. 2011 Oct 11;6(10):e25705. doi: 10.1371/journal.pone.0025705 (PMC3191145; doi:10.1371/journal.pone.0025705)

## Slide 1
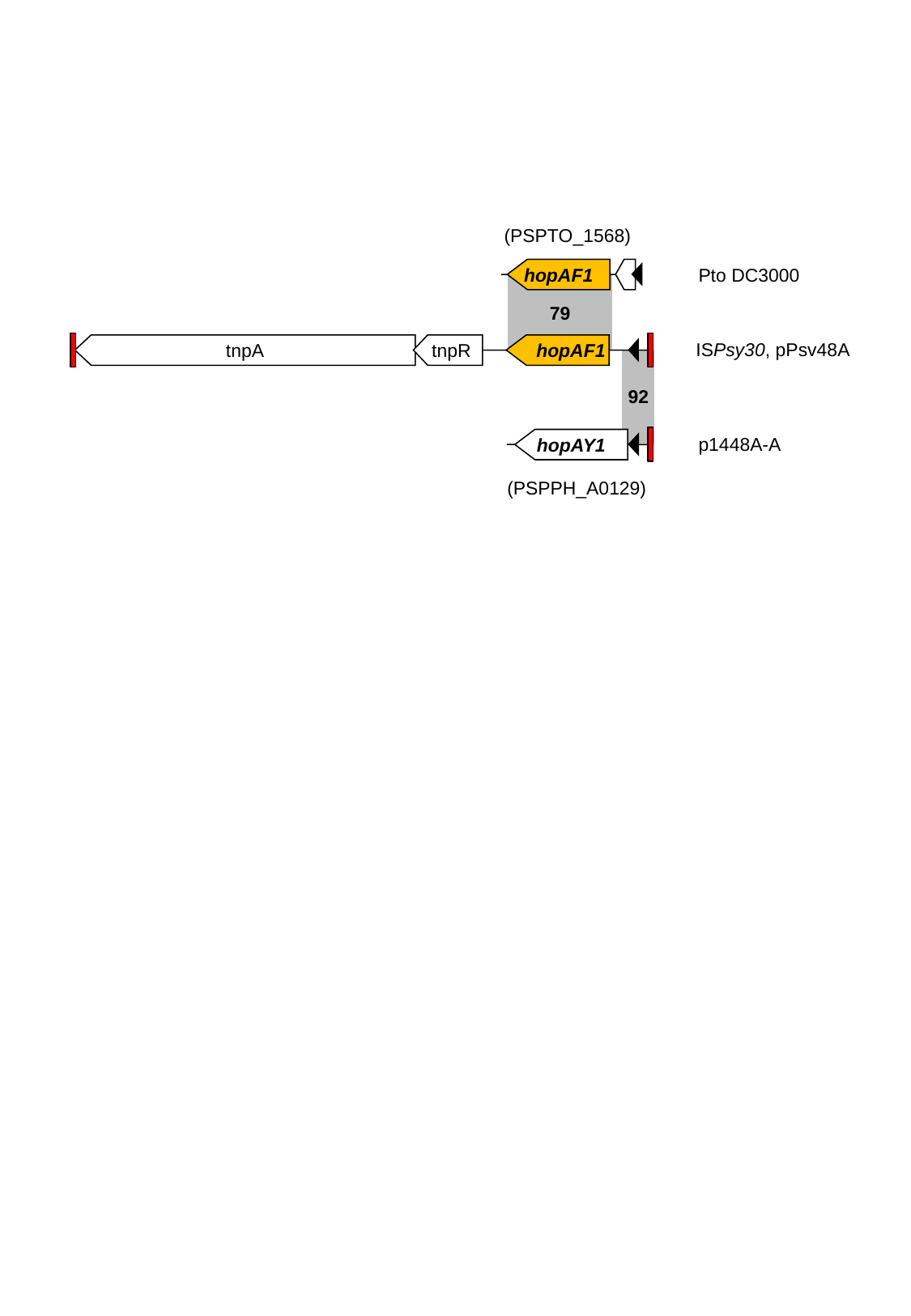

(PSPTO_1568)
Pto DC3000
hopAF1
79
ISPsy30, pPsv48A
tnpA
tnpR
hopAF1
92
p1448A-A
hopAY1
(PSPPH_A0129)

Supplement: Figure S1 — Structure of the effector transposon IS Psy30 found in pPsv48A. Open reading frames are indicated by block arrows, terminal inverted repeats as red rectangles, and hrp boxes as black triangles. Grey bars indicate collinear regions, with the percentage of identity shown. ISPsy30 was compared to the genome of P. syringae pv. tomato DC3000 (accession no. AE016853) and the larger plasmid from P. syringae pv. phaseolicola 1448A (accession no. CP000059). (PPT) [file pone.0025705.s001.ppt]

## Slide 1
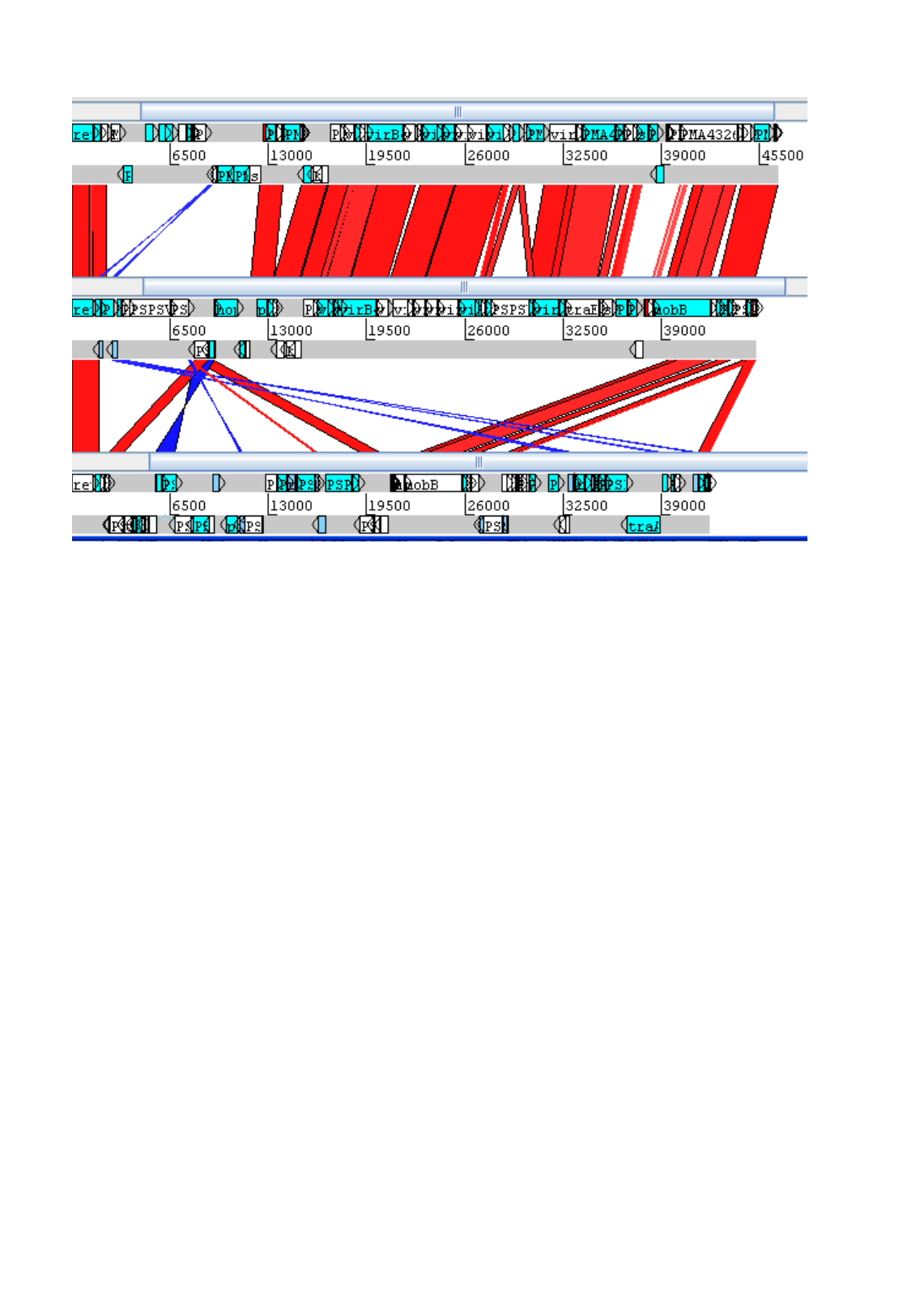

Supplement: Figure S4 — Conservation of plasmids backbone. Pairwise blast alignment of native plasmids pPMA4326A (AY603979; top), pPsv48B (middle) and pPsv48C (bottom), done with WebACT and visualized with ACT; red and blue indicate collinear and inverted regions of identity, respectively.. Only those matches longer than 100 nt with at least 80% identity are shown. (PPT) [file pone.0025705.s004.ppt]

## Slide 1
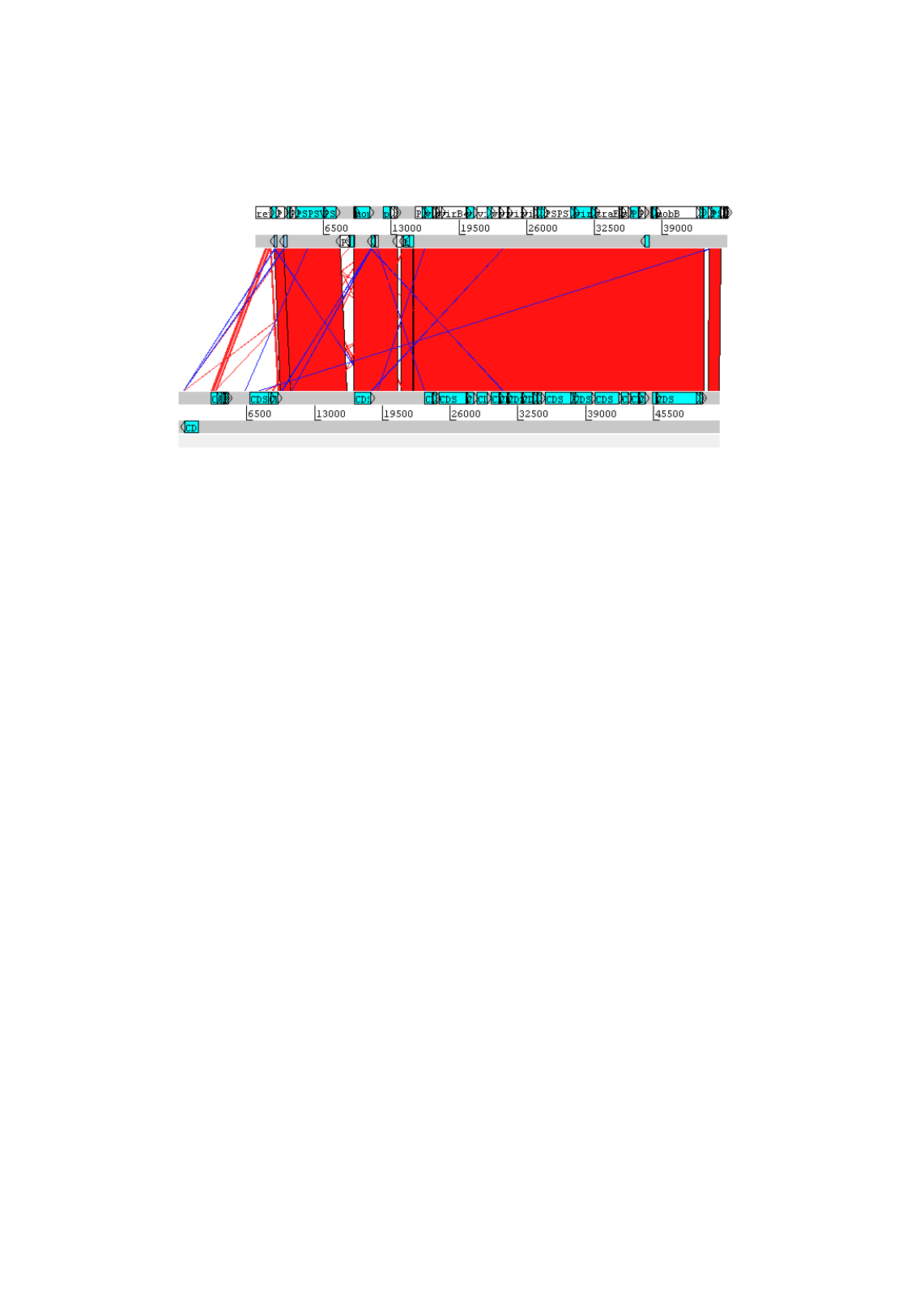

Supplement: Figure S5 — Example of inadequate assembly of plasmid sequences in the draft genome of P. savastanoi pv. savastanoi NCPPB 3335. Comparison of the closed, curated sequence of pPsv48B (upper sequence; 45,220 nt) with supercontig ASAPContig021 (lower sequence; 51,830 nt; https://asap.ahabs.wisc.edu/asap/home.php) obtained after 454 shotgun sequencing and pair-end library analysis. A Blastn comparison was done with WebACT and visualized with ACT; red and blue indicate collinear and inverted regions of identity, respectively. (PPT) [file pone.0025705.s005.ppt]
