# Supplementary material for: Sequence and Role in Virulence of the Three Plasmid Complement of the Model Tumor-Inducing Bacterium Pseudomonas savastanoi pv. savastanoi NCPPB 3335
Source: PLoS One. 2011 Oct 11;6(10):e25705. doi: 10.1371/journal.pone.0025705 (PMC3191145; doi:10.1371/journal.pone.0025705)
Supplement: Figure S2 — Inverted repeats of MITE Psy3 . Comparison of the repeats of MITEPsy3 and the Tn3 family transposon ISThsp9, from Thiomonas sp. Identical nt in at least three sequences are boxed in black. (PPT) [file pone.0025705.s002.ppt]

## Slide 1
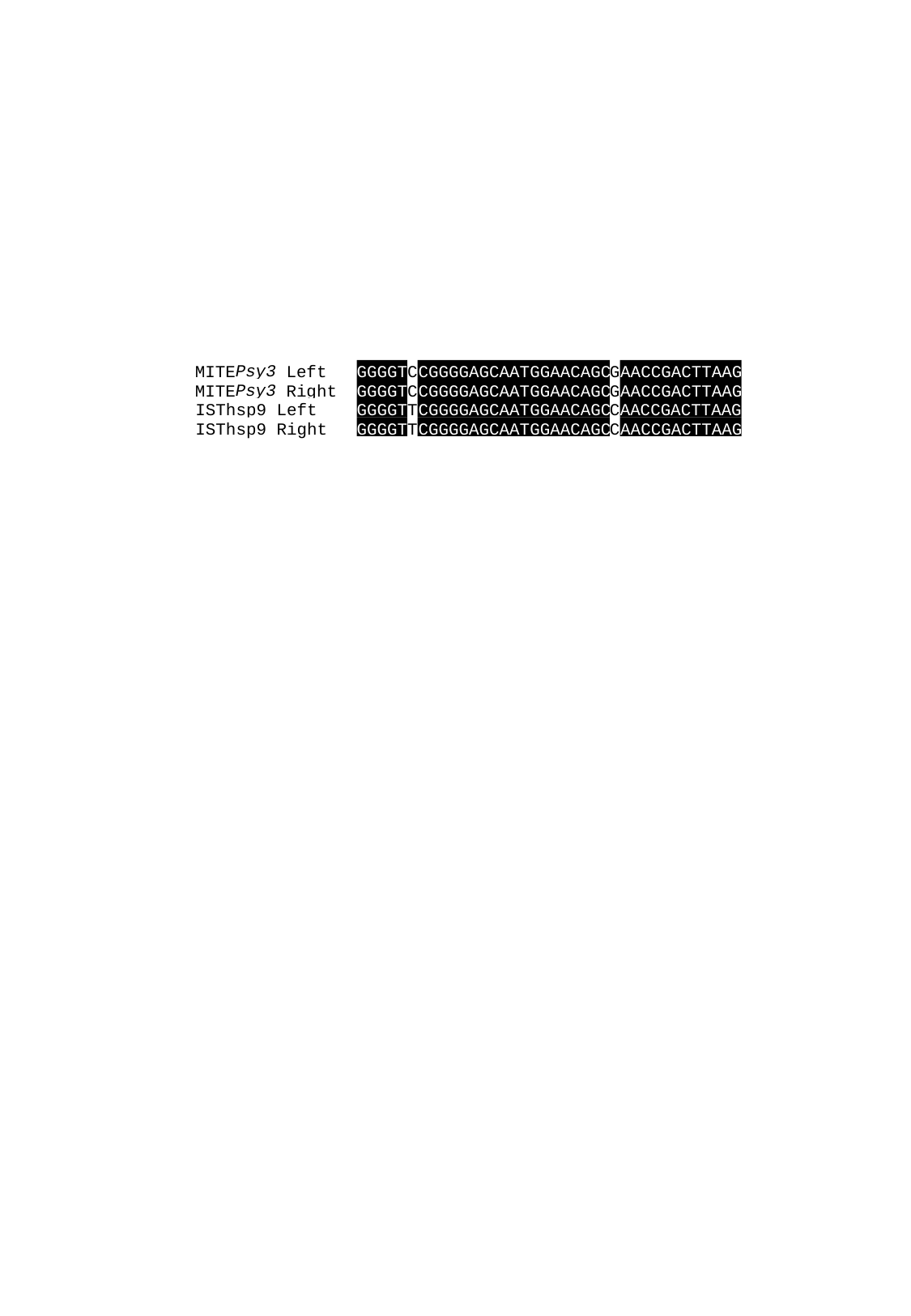

Psy3
MITE
 Left
GGGGT
C
CGGGGAGCAATGGAACAGC
G
AACCGACTTAAG
Psy3
MITE
 Right
GGGGT
C
CGGGGAGCAATGGAACAGC
G
AACCGACTTAAG
ISThsp9 Left
GGGGT
T
CGGGGAGCAATGGAACAGC
C
AACCGA
CTTAAG
ISThsp9 Right
GGGGT
T
CGGGGAGCAATGGAACAGC
C
AACCGACTTAAG
