# Supplementary material for: Sequence and Role in Virulence of the Three Plasmid Complement of the Model Tumor-Inducing Bacterium Pseudomonas savastanoi pv. savastanoi NCPPB 3335
Source: PLoS One. 2011 Oct 11;6(10):e25705. doi: 10.1371/journal.pone.0025705 (PMC3191145; doi:10.1371/journal.pone.0025705)
Supplement: Figure S3 — Phylogenetic analysis of full nucleotide sequences of the repA gene from PFP plasmids from strains of the P. syringae complex. The evolutionary history was inferred by Neighbor-Joining using MEGA5 [69]; evolutionary distances were computed using the Maximum Composite Likelihood method, and pairwise deletion, and are in the units of the number of base substitutions per site; a similar topology was obtained using Maximum parsimony with default settings. The percentages of replicate trees in which the associated taxa clustered together in the bootstrap test (2000 replicates) are shown next to the branches. The tree was constructed with 44 repA sequences previously described [20], plus those from the three plasmids of P. savastanoi pv. savastanoi NCPPB 3335 (arrows) and using the repA from Thiomonas intermedia K12 plasmid pTINT01 (accession no. CP002022, locus tag Tint_3234) as an outgroup; the pathovar of origin of each sequence is shown after the name of the plasmid. Phylogenetic groups are as described [20]; groups A, B and D are shown as triangles proportional to the number of sequences they contain; numbers after the name of groups indicate the genomospecies of the pathovars from which the plasmids were isolated. (PPT) [file pone.0025705.s003.ppt]

## Slide 1
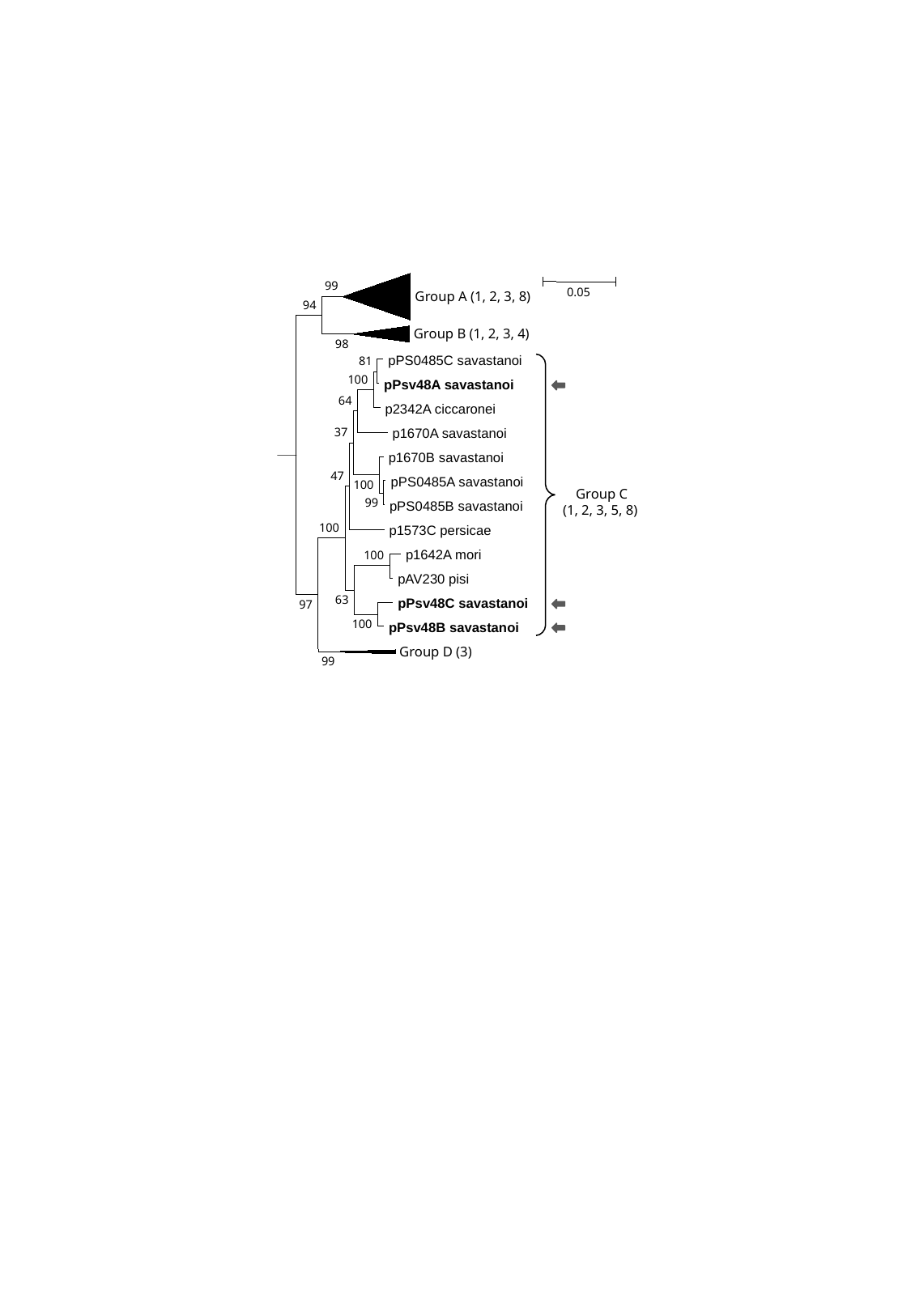

99
 Group A (1, 2, 3, 8)
94
 Group B (1, 2, 3, 4)
98
 pPS0485C savastanoi
81
100
 pPsv48A savastanoi
64
 p2342A ciccaronei
 p1670A savastanoi
37
 p1670B savastanoi
47
 pPS0485A savastanoi
100
 Group C
(1, 2, 3, 5, 8)
99
 pPS0485B savastanoi
100
 p1573C persicae
 p1642A mori
100
 pAV230 pisi
63
 pPsv48C savastanoi
97
100
 pPsv48B savastanoi
 Group D (3)
99
0.05
