# Supplementary material for: Sequence and Role in Virulence of the Three Plasmid Complement of the Model Tumor-Inducing Bacterium Pseudomonas savastanoi pv. savastanoi NCPPB 3335
Source: PLoS One. 2011 Oct 11;6(10):e25705. doi: 10.1371/journal.pone.0025705 (PMC3191145; doi:10.1371/journal.pone.0025705)
Supplement: Table S1 — Number of putative genes predicted in the annotation of the native plasmids of P. savastanoi pv. savastanoi NCPPB 3335, separated by functional categories. (DOC) [file pone.0025705.s006.doc]

| **Table S1.** Number of putative genes predicted in the annotation of the native plasmids of *P. savastanoi* pv. *savastanoi* NCPPB 3335, separated by functional categories. | | | |
| --- | --- | --- | --- |
|  | Plasmid from strain NCPPB 3335 | | |
| Functional category | A | B | C |
| DNA metabolism |  |  |  |
| Replication | 1 | 1 | 2 |
| Toxin/antitoxin genes | 4 | 2 | 5 |
| Other | 13 | 5 | 4 |
| Putative virulence factors | 5 | 1 | 1 |
| Conjugation | 9 | 15 | 5 |
| Transcriptional regulators | 4 | 5 | 3 |
| Transposases | 10 | 2 | 8 |
| Hypothetical | 14 | 14 | 10 |
| Other | - | 5 | 4 |
| Total | 60 | 50 | 42 |
