# Supplementary material for: Sequence and Role in Virulence of the Three Plasmid Complement of the Model Tumor-Inducing Bacterium Pseudomonas savastanoi pv. savastanoi NCPPB 3335
Source: PLoS One. 2011 Oct 11;6(10):e25705. doi: 10.1371/journal.pone.0025705 (PMC3191145; doi:10.1371/journal.pone.0025705)
Supplement: Table S2 — Plasmid features with low (<50%) or high (>62%) G+C content. (DOC) [file pone.0025705.s007.doc]

**Table S2.** Plasmid features with low (<50 %) or high (>62 %) G+C content.

| Plasmid and locus (gene) | Size (nt) | %G+C | Product |
| --- | --- | --- | --- |
| **pPsv48A** |  |  |  |
| PSPSV_A0005 | 7848 | 63.07 | Conserved hypothetical protein |
| PSPSV_A0023 | 903 | 46.17 | Hypothetical protein |
| PSPSV_A0024 (*ptz*) | 705 | 43.40 | Isopentenyl transferase |
| PSPSV_A0025 | 615 | 41.78 | Hypothetical protein |
| PSPSV_A0028 (*hopAF1*) | 843 | 47.21 | Type III effector HopAF1 |
| PSPSV_A0035 | 7848 | 63.27 | Conserved hypothetical protein |
| PSPSV_A0046 | 7092 | 63.18 | Conserved hypothetical protein |
| PSPSV_A0056 (*traW*) | 1221 | 62.24 | Conjugal transfer protein |
| **pPsv48B** |  |  |  |
| PSPSV_B0004 | 273 | 44.68 | Putative arylsulfatase regulatory protein |
| PSPSV_B0013 (*parA*) | 651 | 48.84 | parA/yafB type stability/partitioning protein |
| PSPSV_B0014 | 339 | 47.49 | stability/partitioning protein |
| PSPSV_B0015 | 150 | 49.33 | Hypothetical protein |
| PSPSV_B0016 | 381 | 45.66 | Conserved hypothetical protein |
| PSPSV_B0017 (*bip*) | 471 | 37.36 | Putative bacteriocin immunity protein |
| PSPSV_B0019 | 528 | 41.66 | Putative transcription antiterminator |
| PSPSV_B0020 | 216 | 42.12 | Conserved hypothetical protein |
| PSPSV_B0022 (*virB2*) | 321 | 65.10 | Type IVA conjugal transfer protein |
| PSPSV_B0027 (*virB7*) | 315 | 65.07 | Type IVA conjugal transfer protein |
| PSPSV_B0029 (*virB9*) | 810 | 62.34 | Type IVA conjugal transfer protein |
| PSPSV_B0042 | 735 | 47.61 | Putative stability/partitioning determinant |
| PSPSV_B0043 | 573 | 44.85 | Hypothetical protein |
| **pPsv48C** |  |  |  |
| PSPSV_C0023 | 1674 | 49.4 | Radical SAM domain protein; Putative methyltransferase |
| PSPSV_C0024 (*ipt*) | 459 | 47.71 | Putative isopentenyl-diphosphate delta-isomerase, type 1 |
| PSPSV_C0028 (*mobC*) | 726 | 47.52 | Putative relaxosome component |
| PSPSV_C0038 | 387 | 62.01 | Putative transcriptional regulator |
| PSPSV_C0041 | 279 | 47.31 | Putative arylsulfatase regulatory protein |
| PSPSV_C0043 (*repL*) | 603 | 47.76 | Putative replication protein |
| PSPSV_C0045 | 138 | 46.37 | Hypothetical protein |
| PSPSV_C0046 | 528 | 35.22 | Hypothetical protein |
| PSPSV_C0047 | 1317 | 34.39 | Putative membrane protein |
