# Supplementary material for: Sequence and Role in Virulence of the Three Plasmid Complement of the Model Tumor-Inducing Bacterium Pseudomonas savastanoi pv. savastanoi NCPPB 3335
Source: PLoS One. 2011 Oct 11;6(10):e25705. doi: 10.1371/journal.pone.0025705 (PMC3191145; doi:10.1371/journal.pone.0025705)
Supplement: Table S3 — Genes coding for components of Type IV secretion systems. (DOC) [file pone.0025705.s008.doc]

**Table S3.** Genes coding for components of Type IV secretion systems.

| Plasmid and locus | Psv ID^a^ | Product | Plasmid and locus | Psv ID^a^ | Product |
| --- | --- | --- | --- | --- | --- |
| **pPsv48A** |  |  | **pPsv48B** |  |  |
| PSPSV_A0052 | AER-0000334 | TraT | PSPSV_B0021 | AER-0000609 | VirB1 |
| PSPSV_A0053 | AER-0000335 | TraU | PSPSV_B0022 | AER-0000608 | VirB2 |
| PSPSV_A0056 | AER-0000337 | TraW | PSPSV_B0023 | - | VirB3 |
| PSPSV_A0057 | AER-0000338 | TraX | PSPSV_B0024 | AER-0000607 | VirB4 |
| PSPSV_A0058 | AER-0000339 | TraY | PSPSV_B0025 | AER-0000606 | VirB5 |
| PSPSV_A0059 | AER-0000340 | ExcA | PSPSV_B0026 | AER-0000605 | VirB6 |
| PSPSV_A0060 | AER-0000341 | TrbA | PSPSV_B0027 | - | VirB7 |
| PSPSV_A0061 | AER-0000342 | TrbB | PSPSV_B0028 | AER-0000604 | VirB8 |
| PSPSV_A0062 | AER-0000343 | TrbC | PSPSV_B0029 | AER-0000603 | VirB9 |
|  |  |  | PSPSV_B0030 | AER-0000602 | VirB10 |
| **pPsv48C** |  |  | PSPSV_B0031 | AER-0000601 | VirB11 |
| PSPSV_C0027 | - | TraD | PSPSV_B0037 | AER-0000596 | VirD4 |
| PSPSV_C0028 | AER-0003633 | MobC | PSPSV_B0039 | AER-0000595 | TraE |
| PSPSV_C0029 | AER-0003632 | MobB | PSPSV_B0045 | AER-0000591 | MobC |
| PSPSV_C0048 | AER-0002647 | TraA | PSPSV_B0046 | AER-0000590 | MobB |
| PSPSV_C0049 | AER-0002646 | MobC |  |  |  |

^a^ ASAP ID number in the draft genome sequence of *P. savastanoi* pv. savastanoi strain NCPPB 3335. (-) means that the gene was not annotated in the genome sequence.
