# Supplementary material for: Sequence and Role in Virulence of the Three Plasmid Complement of the Model Tumor-Inducing Bacterium Pseudomonas savastanoi pv. savastanoi NCPPB 3335
Source: PLoS One. 2011 Oct 11;6(10):e25705. doi: 10.1371/journal.pone.0025705 (PMC3191145; doi:10.1371/journal.pone.0025705)
Supplement: Table S4 — Putative virulence genes found in the native plasmids from P. savastanoi pv. savastanoi NCPPB 3335. (DOC) [file pone.0025705.s009.doc]

**Table S4.** Putative virulence genes found in the native plasmids from *P. savastanoi* pv. savastanoi NCPPB 3335.

|  |  |  |  |  | Highest identity | | |
| --- | --- | --- | --- | --- | --- | --- | --- |
| Plasmid and locus | Psv ID^a^ | Size in nt (G+C %) | hrp box | Product | Locus | Global % aa identity^b^ | E value, Organism |
| **pPsv48A** |  |  |  |  |  |  |  |
| PSPSV_A0005 | AER-0003949 | 7848 (63.07) | yes | Conserved hypothetical protein | PSPPH_1525 | 91 | 0; *P. syringae* pv. phaseolicola 1448A |
| PSPSV_A0024 | - | 705 (43.4) | no | Ptz, isopentenyl transferase | IPT_PSESS | 99.6 | 4e-135; *P. savastanoi* pv. nerii 1006 |
|  |  |  |  |  | XALc_2569 | 49.2 | 1e-58; *Xanthomonas* *albilineans* GPE PC73 |
|  |  |  |  |  | IPT_AGRRH | 46.3 | 1e-56; *Agrobacterium* *rhizogenes* A4 |
| PSPSV_A0028 | AER-0003643 | 843 (47.21) | yes | type III effector HopAF1 | PSPPH_1443 | 73.6 | 2e-119; *P. syringae* pv. phaseolicola 1448A |
|  |  |  |  |  | AER-0000968 | 73.6 | 1e-118, *P. savastanoi* pv. savastanoi NCPPB 3335 |
| PSPSV_A0035 | - | 7848 (63.27) | yes | Conserved hypothetical protein | PSPPH_1525 | 91 | 0; *P. syringae* pv. phaseolicola 1448A |
| PSPSV_A0046 | - | 7092 (63.18) | yes | Conserved hypothetical protein | PSPPH_1525 | 84.2 | 0; *P. syringae* pv. phaseolicola 1448A |
| **pPsv48B** |  |  |  |  |  |  |  |
| PSPSV_B0008 | AER-0000610 | 1398 (51.57) | yes | Type III effector HopAO1 | Psyrpa2_010100025464 | 98.1 | 0; *Pseudomonas* *syringae* pv. aesculi 2250 |
| **pPsv48C** |  |  |  |  |  |  |  |
| PSPSV_C0024 | - | 459 (47.71) | no | Ipt, isopentenyl-diphosphate delta-isomerase, type 1 | PROSTU_01104 and PROSTU_04490 | 43.9 | 8e-35; *Providencia* *stuartii* ATCC 25827 |

^a^ ASAP ID number in the draft genome sequence of *P. savastanoi* pv. savastanoi strain NCPPB 3335. (-) means that the gene was not annotated in the genome sequence.

^b^ Identity calculated using the program EMBOSS Align.
