# Supplementary material for: Sequence and Role in Virulence of the Three Plasmid Complement of the Model Tumor-Inducing Bacterium Pseudomonas savastanoi pv. savastanoi NCPPB 3335
Source: PLoS One. 2011 Oct 11;6(10):e25705. doi: 10.1371/journal.pone.0025705 (PMC3191145; doi:10.1371/journal.pone.0025705)
Supplement: Table S5 — Type and number of mobile elements found in the native plasmids of P. savastanoi pv. savastanoi NCPPB 3335. (DOC) [file pone.0025705.s010.doc]

| **Table S5.** Type and number of mobile elements found in the native plasmids of *P. savastanoi* pv. savastanoi NCPPB 3335^a^ | | | | |
| --- | --- | --- | --- | --- |
| Mobile element | Size (nt) | pA | pB | pC |
| CRR1 | 1752-1765 | 4 |  | 1 |
| IS*51* (syn. IS*Psy21*) | 1312 | 4 | 1 | 2 (1) |
| IS*53* (syn. IS*Psy20*) | 2570 | (1) |  |  |
| IS*Ppa26* |  |  |  | (1) |
| IS*1240* | 1662 |  |  | (1) |
| IS*Psy2* |  |  |  | (1) |
| IS*Psy16* | 1461 | (1) |  | 2 |
| IS*Psy24* | 1235 |  |  | (1) |
| IS*Psy30*^b^ | 5167 | 1 |  |  |
| IS*Shes11* | 7669 |  |  | (1) |
| TnpA2 | 540 |  |  | 1 |
| MITE*Psy1*^c^ | 100 |  |  | 1 |
| MITE*Psy2* | 228 | 1 | 1 (1) | 2 |
| MITE*Psy3* | 334 |  |  | 1 |
|  |  |  |  |  |
| Combined size (nt) |  | 19399 | 1665 | 12439 |

^a^ Numbers in parenthesis indicate degenerate elements.

^b^ The configuration and gene content of this insertion sequence is variable among copies in different organisms; in pPsv48A, it includes the effector gene *hopAF1* in a total of 5167 nt.

^c^ MITE*Psy1* was shown to actively transpose in *P. syringae* pv. phaseolicola [37].
