# Supplementary material for: Sequence and Role in Virulence of the Three Plasmid Complement of the Model Tumor-Inducing Bacterium Pseudomonas savastanoi pv. savastanoi NCPPB 3335
Source: PLoS One. 2011 Oct 11;6(10):e25705. doi: 10.1371/journal.pone.0025705 (PMC3191145; doi:10.1371/journal.pone.0025705)
Supplement: Table S6 — Bacterial strains and plasmids used in this work. (DOC) [file pone.0025705.s011.doc]

| **Table S6.** Bacterial strains and plasmids used in this work. | | |
| --- | --- | --- |
| Strains and plasmids | Characteristics | Source or reference^a^ |
| **Bacterial strains** |  |  |
| *Escherichia* *coli* |  |  |
| NEB 5-alpha | Derivative of DH5α; *fhuA2* Δ(*argF-lacZ*)*U169 phoA glnV44* Φ*80*Δ (*lacZ*)M15 *gyrA96 recA1 relA1 endA1 thi-1 hsdR17* | NEB |
| XL1 blue | *recA1 endA1 gyrA96 thi-1 hsdR17 supE44 relA1 lac* [F´ *proAB lacI*q*Z*Δ*M15* Tn*10* (Tet^R^)] | Stratagene |
| *Pseudomonas* *fluorescens* | |  |
| SBW25 | Wild type | [1] |
| *P. putida* |  |  |
| KT2440 | *hsdR* | [2] |
| *P.* *savastanoi* pv. savastanoi | |  |
| NCPPB 3335 | Wild type strain; synonymous of Psv48 | NCPPB^a^ |
| Psv48ΔA | Psv48 cured of pPsv48A | This work |
| Psv48ΔAB | Psv48 cured of pPsv48A and pPsv48B | This work |
| *P. syringae* pv. syringae | |  |
| B728a | Wild type; Rif^R^, Cu^R^, str^R^ | [3] |
| **Plasmids** |  |  |
| pBluescript SK II | Commercial cloning vector Amp^R^ | Stratagene |
| pGEM-3Z | Commercial cloning vector Amp^R^ | Promega |
| pGEM-T Easy | Commercial cloning vector Amp^R^ | Promega |
| pCR2.1 | Commercial cloning vector Amp^R^; Km^R^ | Invitrogen |
| pDR1 | Delivery vector for Tn*5*-GDYN1, based on pSUP2021; Km^R^, Gm^R^, confers sucrose-dependent lethality | [4] |
| pPsv48A::Tn*5*-GDYN1 | pPsv48A containing an insertion of Tn*5*-GDYN1; Km^R^, Gm^R^, confers sucrose-dependent lethality | This work |
| pPsv48C::Tn*5*-GDYN1 | pPsv48C containing an insertion of Tn*5*-GDYN1; Km^R^, Gm^R^, confer sucrose-dependent lethality | This work |
| pLRM1-GFP | pBBR1-MCS5 carrying a fusion of the P_A1/04/03_ promoter to the *gfpmut3** gene | [5] |
|  | | |

^a^ NEB, New England Biolabs (Ipswich, MA); NCPPB, National collection of Plant Pathogenic bacteria, York , United Kingdom.

References

1. Rainey PB, Bailey MJ (1996) Physical and genetic map of the *Pseudomonas fluorescens* SBW25 chromosome. Molecular Microbiology 19: 521-533.

2. Franklin FC, Bagdasarian M, Bagdasarian MM, Timmis KN (1981) Molecular and functional analysis of the TOL plasmid pWWO from *Pseudomonas putida* and cloning of genes for the entire regulated aromatic ring meta cleavage pathway. Proceedings of the National Academy of Sciences USA 78: 7458-7462.

3. Loper JE, Lindow SE (1987) Lack of evidence for *in situ* fluorescent pigment production by *Pseudomonas syrignae* pv. *syringae* on bean leaf surfaces. Phytopathology 77: 1449-1454.

4. Flores M, Brom S, Stepkowski T, Girard ML, Dávila G, et al. (1993) Gene amplification in *Rhizobium*: identification and *in vivo* cloning of discrete amplifiable DNA regions (amplicons) from *Rhizobium leguminosarum* biovar *phaseoli*. Proceedings of the National Academy of Science USA 90: 4932-4936.

5. Rodríguez-Moreno L, Jiménez AJ, Ramos C (2009) Endopathogenic lifestyle of *Pseudomonas savastanoi* pv. *savastanoi* in olive knots. Microbial Biotechnology 2: 476-488.
